# Towards understanding centriole elimination

**DOI:** 10.1098/rsob.230222

**Published:** 2023-11-15

**Authors:** Nils Kalbfuss, Pierre Gönczy

**Affiliations:** Swiss Institute for Experimental Cancer Research (ISREC), School of Life Sciences, Swiss Federal Institute of Technology Lausanne (EPFL), 1015 Lausanne, Switzerland

**Keywords:** centriole maintenance, centriole elimination, centriole repair, organelle degradation

## Abstract

Centrioles are microtubule-based structures crucial for forming flagella, cilia and centrosomes. Through these roles, centrioles are critical notably for proper cell motility, signalling and division. Recent years have advanced significantly our understanding of the mechanisms governing centriole assembly and architecture. Although centrioles are typically very stable organelles, persisting over many cell cycles, they can also be eliminated in some cases. Here, we review instances of centriole elimination in a range of species and cell types. Moreover, we discuss potential mechanisms that enable the switch from a stable organelle to a vanishing one. Further work is expected to provide novel insights into centriole elimination mechanisms in health and disease, thereby also enabling scientists to readily manipulate organelle fate.

## Graphical abstract

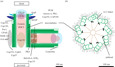

## 1. Foreword

Centrioles are evolutionarily conserved organelles that serve a range of fundamental cellular functions, including motility, signalling and division. Although the centriole organelle exhibits exceptional stability, it can be eliminated in some settings, as was already recognized in the case of the oocyte by the pioneering work of Theodor Boveri [1]. More instances of centriole elimination have been uncovered since, but a general understanding of the underlying mechanisms is lacking, precluding an assessment of the potential function of such disappearance in most cases. In this review, we first discuss cases of centriole elimination in several systems, then cover potential mechanisms contributing to organelle maintenance and elimination, before mentioning some directions for the future.

## 2. Introduction

**Figure 1.**
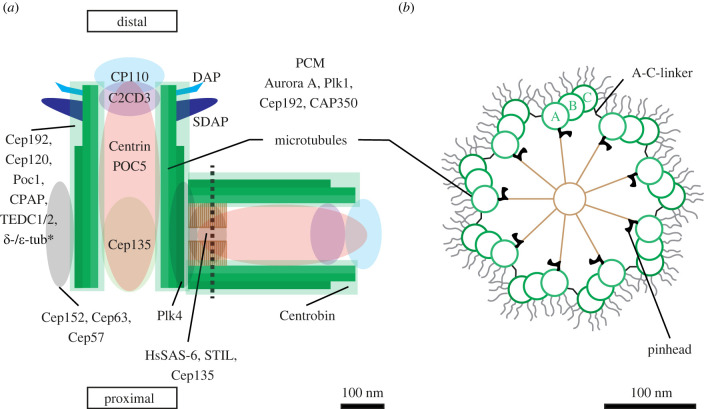
Schematic of human centriole and procentriole, with approximate distribution of proteins discussed in this review. (*a*) Longitudinal section of mother centriole (left), distinguishable by the presence of subdistal and distal appendages (SDAP and DAP, respectively), and accompanying procentriole (right), with cartwheel (brown). Dashed line indicates the position of the cross-section shown in (*b*). Approximate locations of select centriolar and PCM components are indicated by shaded regions in (*a*). Note that this schematic does not represent detailed distributions that have been revealed for some of these proteins using super-resolution and expansion microscopy. In general, similarly shaded colours on the mother centriole and the procentriole indicate that the same set of proteins is present in those locations. Centrobin localizes to the microtubule wall solely on the procentriole. Note that the C-termini of tubulins, which are represented in (*b*), are not shown in (*a*). Note also that although δ- and ε-tubulin localize to centrosomes [2] and are needed for microtubule doublet/triplet formation [3], their exact localization is not known (indicated by *). Finally, note that Plk4 is present initially throughout the torus, before focusing onto a single site below the incipient procentriole. (*b*) Corresponding cross-section of procentriole viewed from the distal end. A characteristic feature of centrioles is their 9-fold radially symmetrical microtubule array, which is largely conserved across species, although there are interesting variations (reviewed by [4]). The proximal side of the centriole harbours microtubule triplets, dubbed A-, B- and C-microtubules, whereas the distal side bears only A- and B-microtubule doublets. When viewed from the distal end (*b*), microtubule triplets are arranged in a clockwise fashion, yielding a chiral structure (reviewed by [4,5]). In the proximal side, the A-microtubule of a given triplet is connected with the C-microtubule of the adjacent triplet by the A–C linker, giving the organelle a continuous outer wall. The proximal-most approximately 100 nm of the procentriole bears the cartwheel, which consists of a central hub from which emanate 9 spokes that then connect to peripheral microtubules through the pinhead (reviewed by [6]).

Centrioles are barrel-shaped microtubule-containing organelles typically approximately 500 nm × 250 nm in dimensions located in the vicinity of the nucleus in cycling cells (figure 1) (reviewed by [7–10]). In many terminally differentiated cells, centrioles dock below the plasma membrane, where they act as basal bodies that template the axoneme of the primary cilium (reviewed by [11]). Centrioles also template the axoneme of motile cilia and flagella in cells bearing these structures. Through these roles, centrioles are essential for signal transduction and cell movement (reviewed by [11]). Moreover, in most cycling animal cells, centrioles are embedded in the pericentriolar material (PCM, also referred to as pericentriolar matrix), with which they constitute the centrosome, a major microtubule organizing centre (MTOC). Through this role, centrioles are important for cellular organization during interphase, as well as during mitosis, when the two centrosomes present at that stage of the cell cycle ensure bipolar spindle assembly and faithful chromosome segregation (reviewed by [12]). As may be anticipated from such important physiological functions, alterations in centriole structure or number can contribute to pathologies, including ciliopathies and cancer (reviewed by [13–15]).

Centriole number is tightly regulated. Cycling cells initially harbour a so-called mother centriole, characterized by distal and subdistal appendages, and a so-called daughter centriole, which is connected to the mother centriole via a flexible linker. Approximately at the onset of S phase, a procentriole forms near-orthogonal to each pre-existing centriole. During G2/early mitosis, the two pairs of centriole/procentriole, each surrounded by PCM and thereby constituting a centrosome, separate from one another and direct bipolar spindle assembly. The proteins and mechanisms ensuring orderly progression through this centriole duplication cycle are well characterized (reviewed by [16–19]) (box 1).

Box 1. **Centriole assembly.**

The core centriole assembly pathway is largely conserved among eukaryotic species. We summarize hereafter how this pathway operates in human cells (see also figure 1). Each newly born cell is endowed with one older (mother) and one younger (daughter) centriole, the latter having been formed in the previous cell cycle (reviewed by [20]). The presence of subdistal and distal appendages, which are acquired during G2 and/or M phase by the younger centriole [21], allows one to distinguish mature mother centrioles from daughter centrioles [5]. An important element at the onset of centriole biogenesis is a torus that encircles the proximal third of each pre-existing centriole, and which comprises a complex of the proteins Cep152-Cep63-Cep57 [22–29]. The Polo-like-kinase 4 (Plk4) is recruited to this torus at the onset of the assembly process. Plk4 homodimerization leads to trans-autophosphorylation and subsequent SCF-dependent proteasome-mediated degradation of the kinase [30–36]. Plk4 also phosphorylates STIL [37–40], which binds Plk4 and protects it from degradation, thereby eventually leading to the focusing of the two proteins to a single location on the torus. STIL also binds HsSAS-6 [38–40], the key building block of the so-called cartwheel that forms at the site where Plk4 and STIL enrich [41,42]. Across species, SAS-6 proteins harbour a coiled-coil domain that mediates homodimerization, as well as a globular N-terminal head-domain that drives association between SAS-6 homodimers. Such association results in the formation of ring-containing structures comprising 9 SAS-6 homodimers [41,42], which stack to form the cartwheel. The cartwheel is connected to the peripheral microtubules by the so-called pinhead, which has been proposed to contain notably Cep135 [43–46]. Plk4-mediated phosphorylation of STIL also promotes STIL binding to CPAP, thereby facilitating the incorporation of CPAP into the growing procentriole [47–49]. Together with Cep120, Poc1 and CP110, CPAP enables procentriole elongation [50–55]. More components are recruited thereafter, including Centrin, which is present in the central core and the distal lumen of the organelle [56–60].

**Figure 2.**
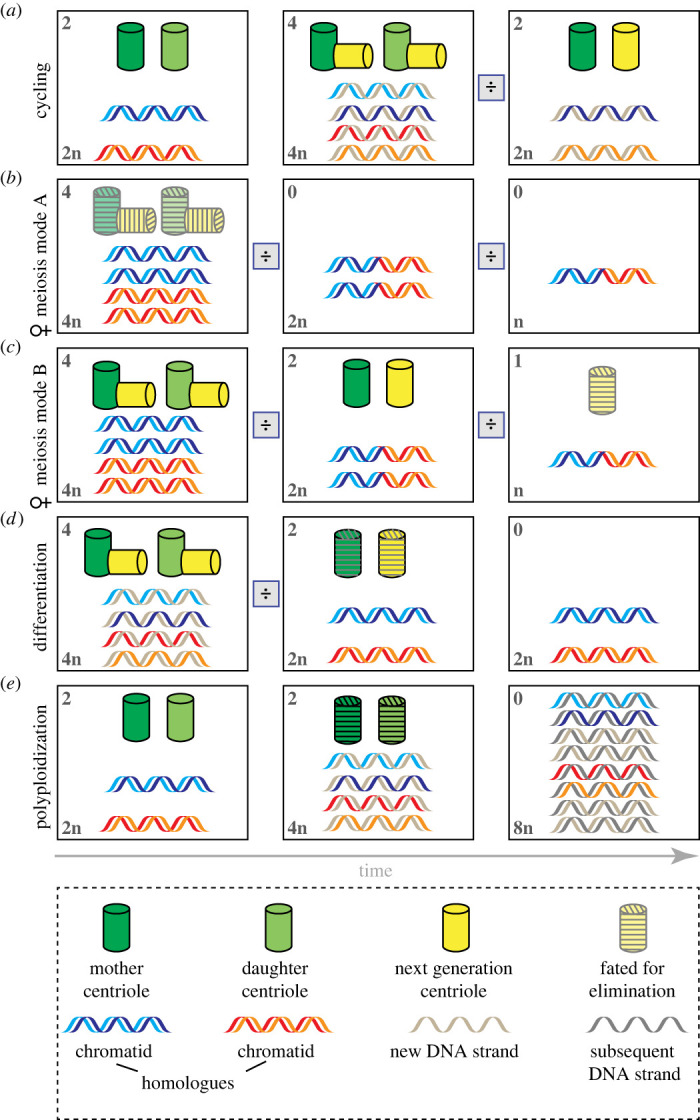
Schematic comparison of centriole and DNA numbers in different settings. (*a*) Cycling cells are born with 2 homologous chromatids, as well as so-called mother and daughter centrioles. DNA and centrioles are both copied during S phase. Whereas DNA replication is semi-conservative, with a newly synthesized strand and an older strand together forming a chromosome, centriole duplication is conservative, with a procentriole generated anew in the vicinity of each pre-existing centriole. (*b,c*) During female meiosis, following the two pre-meiotic S phases, DNA and centriole numbers are reduced to prepare the oocyte for fusion with a sperm cell containing one set of chromatids and usually two centrioles. (*b*) In a first mode of female meiosis (mode A), encountered for instance in flies, worms and humans, centrioles are eliminated before meiosis and, therefore, before DNA reduction. (*c*) In a second mode of female meiosis (mode B), exemplified by starfish oocytes, together with DNA, two centrioles are extruded into the first polar body and one centriole into the second polar body, leaving in the cytoplasm of the zygote just one centriole, which is subsequently eliminated. (*d*) In terminally differentiated cells, DNA replication no longer takes place, and centriole elimination occurs in some cases. (*e*) During polyploidization, there are multiple rounds of DNA replication, but new centrioles do not necessarily form and can even be eventually eliminated. Numbers on the top left indicate centriole numbers, those on the bottom left ploidy. ÷ indicates mitotic (*a,d,e*) or meiotic (*b,c*) divisions.

Whereas centriole number increases from 2 to 4 in cycling cells (figure 2*a*), centriole number control differs in other settings. For instance, some cells are devoid of centrioles to start with and then form them de novo (reviewed in [61]). This is the case for example in early rodent embryos or in some plant groups, including bryophytes, pteridophytes and gymnosperms, as well as in the excavate *Naegleria gruberi* upon amoeboid to flagellate transformation [62–66]. Moreover, centrioles form de novo in human cells experimentally depleted of the organelle [62–66]. Furthermore, in multiciliated epithelial cells, several procentrioles assemble around pre-existing centrioles and so-called deuterosomes, thereby rapidly amplifying centriole number to one hundred or more [67–70]. Finally, centriole number can also decrease from four or two to none (figure 2*b–e*). This process is referred to as centriole elimination and is the focus of this review.

## 3. Centriole elimination in the female germline

With the advent of electron microscopy (EM) in the 1950s came the first observation of the beautiful signature 9-fold radially symmetric arrangement of centriolar microtubules [71,72]. Further ultrastructural analysis uncovered that centrioles are absent from vertebrate oocytes [73,74], as anticipated from the initial observations of Boveri in sea urchin eggs [1]. Centriole elimination during oogenesis is now recognized as a widespread phenomenon that occurs throughout metazoan organisms.

Despite such widespread occurrence, the timing and mode of oogenesis centriole elimination differ between systems. In a first mode occurring for instance in *X. laevis*, *M. musculus* and *H. sapiens*, as well as in *C. elegans* and *Drosophila*, centrioles are eliminated during the prolonged prophase of meiosis I, resulting in acentriolar meiotic spindles [75,76] (reviewed by [77]) (figure 2*b*). Recent correlative light electron microscopy (CLEM) analysis in *C. elegans* revealed that centrioles lose the so-called central tube in late pachytene, which is followed by the lack of a recognizable centriole at the beginning of diplotene [78]. In *Drosophila*, the maturing oocyte is first endowed with a cluster containing many centrioles contributed by the 15 supporting nurse cells [79,80]. This cluster is important for the transport of mRNAs and proteins from nurse cells to the oocyte and remains present until just before meiotic spindle assembly, although overall centriole number within the cluster seems to decrease already before [79,80]. Thereafter, centriole elimination entails departure of the Polo kinase from the PCM, followed by PCM loss and ultimately organelle elimination [81].

**Figure 3.**
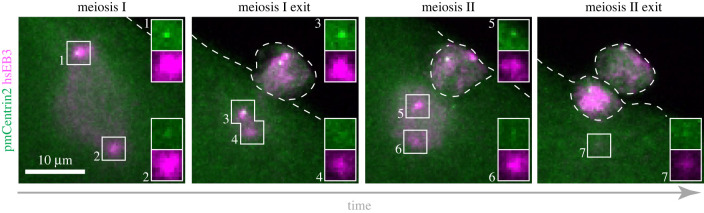
Oocyte centriole elimination in the starfish *P. miniata.* Sequence of events in starfish oocytes expressing *P. miniata* mEGFP::Centrin-2 to mark centrioles, and human EB3::mCherry3 to mark microtubules. In meiosis I, each spindle pole comprises two centrioles, such that two centrioles are extruded in the first polar body. In meiosis II, a mother centriole constitutes the outer meiotic spindle pole, which is extruded into the second polar body. Only one daughter centriole remains in the zygote, which is rapidly eliminated in the fertilized egg. Modified with permission from [82].

In a second mode occurring in echinoderms, mollusks and annelids, centrioles are not eliminated during oogenesis, but instead are removed during and subsequent to the female meiotic division (reviewed in [77]) (figures 2*c* and 3). In these species, as a result of centrioles being located at spindle poles, three of the four initial centrioles are extruded into the polar bodies during the two meiotic divisions, leaving a single centriole in the oocyte proper [83–86]. In the starfish *P. miniata*, this remaining organelle is invariably a daughter centriole, whereas the two mother centrioles and one daughter centriole are extruded into the polar bodies [87]. Also in this case, as in *Drosophila*, the daughter centriole first sheds the surrounding PCM and vanishes subsequently, after sperm centrioles have already recruited PCM components [87].

An intermediate situation compared to the above two modes occurs in the snail *Lymnea stagnalis*. Here, maturing oocytes are endowed with only one pair of centrioles [88]. During meiosis I, the two spindle poles each harbour one centriole, such that one is extruded into the first polar body. During meiosis II, the sole remaining maternal centriole is positioned outward and therefore subsequently extruded into the second polar body, whereas the inward spindle pole is organized by the two sperm-derived centrioles [88]. Furthermore, in the androgenetic clam *Corbicula leana*, the sole meiotic spindle yields two polar bodies that contain the complete maternal DNA complement and four centrioles, with the sperm subsequently restoring DNA and centriole contents [89]. The above examples illustrate that there is substantial diversity in how systems ensure that no functional centriole is left in the oocyte.

## 4. Centriole reduction in the male germline

Centriole reduction, and sometimes elimination, can also occur during male gametogenesis. Indeed, whereas sperm cells often contribute two full-fledged centrioles to the zygote, including in *C. elegans*, sea urchin or starfish [90–93] (reviewed by [77]), this is not always the case. In human sperm, for example, the distal centriole that templates the flagellar axoneme degenerates during the course of spermatogenesis, with microtubule triplets being disassembled, whereas the centriole proximal to the nucleus remains largely intact [94] (reviewed by [77,95]). Some centriolar proteins remain in a focus at the location of the former distal centriole, including POC1B, CETN1/2, POC5 and CPAP [96]. Both distal and proximal sperm-derived entities are thought to be functional in the zygote, as evidenced by their competence to recruit centrosomal components in *Xenopus* extracts [96].

Another interesting example is encountered in *Drosophila*. Here, a giant centriole (GC) that templates the axoneme and a degenerate proximal centriole like structure (PCL) are present in mature sperm [97–99]. Whereas the GC maintains a microtubule wall, the PCL does not [97,99]. Moreover, many centriolar proteins, including Asl, Ana1, Bld10, Ana2, Sas6 and Sas4, are lost from both centrioles by the end of spermatogenesis [98]. By contrast, Poc1B remains at the GC and is enriched in the PCL [99]. Both GC and PCL are functional in the zygote, as they each seed recruitment of the PCM component Asl, as well as of the centriolar proteins Sas6 and Sas4 [98], echoing the findings in human sperm. Interestingly, loss of Asl from mature sperm is essential for robust sperm aster formation after fertilization [100]. Nevertheless, Asl must be recruited from the maternal protein pool to form such sperm asters [100]. Similar to the *Drosophila* case, bovine sperm contributes one canonical and one degenerate centriole, which each recruits PCM components and SAS-6 [96,101,102]. Ultrastructural analysis of bovine embryos further demonstrates that atypical centrioles present in the early embryo can seed procentriole formation [103]. Overall, these cases show that sperm-derived centrioles can degenerate in some cases, while retaining important functions (box 2).

Box 2. **On the hurdles of monitoring a minuscule and vanishing organelle.**

*Stricto sensu*, centrioles are defined at the ultrastructural level by the signature 9-fold symmetric arrangement of microtubules (figure 1). However, centrioles can also be defined at the functional level as entities able to recruit PCM and to template axoneme formation. In this review, we define centriole elimination as the process whereby a centriole loses both ultrastructure and function. Given their tiny dimensions and low copy number in most cells, the presence or absence of centrioles has been evaluated often through the monitoring of a focus bearing centriolar proteins rather than by lower throughput electron microscopy (EM) approaches. In some cases, including in human cells upon depletion of RBM14, Neurl4 or TRIM37, foci bearing centriolar proteins that act as MTOCs have been revealed by EM analysis not to harbour microtubules [104–108]. These examples illustrate that foci bearing centriolar proteins do not necessarily correspond to bona fide centrioles, despite retaining their function in recruiting PCM and acting as MTOCs. By extension, during centriole elimination, perhaps foci of centriolar proteins can likewise be present and recruit PCM, despite the signature centriolar microtubules having vanished. Obviously, monitoring several centriolar proteins reduces the risk of being misled in such cases. Moreover, expansion microscopy methods now enable resolutions that are sufficient to monitor the signature 9-fold radially symmetrical distribution of microtubules [109,110], and are likely to become a standard means to ascertain with reasonable throughput whether foci bearing centriolar proteins also harbour centriolar microtubules.

Rodents represent an extreme case of centriole reduction during spermatogenesis as both centrioles degenerate completely by the end of gametogenesis, despite retention of foci bearing the core centriolar protein Centrin [62,64] (box 2). The absence of centrioles from both oocyte and sperm results in acentriolar cell divisions in the early embryo, which is followed by de novo centriole formation at the blastocyst stage [62,64].

## 5. How frequent is centriole elimination?

Centriole elimination is by no means restricted to germ cells. Indeed, many instances of organelle removal have been described also in somatic cells across the eukaryotic tree of life (table 1). Notably, centriole elimination occurs in many cell types upon terminal differentiation (figure 2*d*), which has been documented in particular in *C. elegans* and *Drosophila*. In general, it is thought that centrioles are present in the many terminally differentiated cells that bear a primary cilium, where centrioles act as basal bodies that template the ciliary axoneme. However, centrioles in *C. elegans* sensory neurons degenerate after having initiated axoneme assembly [123], leaving merely focused PCM components at the ciliary base [130,131], indicating that even ciliated cells can dispose of centrioles in some instances.

**Table 1.** Occurrence of centriole elimination in different species. A check mark in parentheses signifies that this is the case at least for some cells.

species	cell type	syncitium	polyploidization	fusion	references
*N. gruberi*	unicellular	—	—	—	Dingle & Fulton [111]; Fulton & Dingle [112]
*D. melanogaster*	nurse cells	✓	✓	—	Mahowald & Strassheim [113]
	follicle cells	—	✓	—	Mahowald *et al*. [114]
	eye (ommatidial cells, interommatidial cells)				Riparbelli *et al*. [115]
	midgut enterocytes, salivary gland secretory cells, polyploid enterocytes of the hindgut ileum, Malpighian tubule cells		✓		Schoenfelder *et al*. [116]
	fat body cells		✓		Zheng *et al*. [117]
	wing epidermal cells				Morgensen & Tucker [118]
*H. sapiens; M. musculus; G. gallus*	myotubes	✓		✓	Tassin *et al*. [119]; Bugnard *et al*. [120]; Connolly *et al.* [122]
*G. gallus; M. musculus*	skeletal myofibres		✓		Przybylski [121]
*C. elegans*	myotubes	✓		✓	Connolly *et al*. [122]
	sensory neurons	—	—	—	Serwas *et al*. [123]
	intestinal cells	(✓)	✓	—	Lu & Roy [124]; Kalbfuss *et al*. [125]
	seam cells	✓	—	✓	Lu & Roy [124]; Kalbfuss *et al*. [125]
	vulE and vulF	✓		✓	Lu & Roy [124]
	muscle cells, neurons	—	—	—	Kalbfuss & Gönczy [126]
	hypodermal (embryonic)	(✓)	—	✓	Kalbfuss & Gönczy [126]
	hypodermal (larval)	✓	✓	✓	Kalbfuss *et al*. [125]
	pharyngal	(✓)	—	✓	Kalbfuss & Gönczy [126]
	majority of other cell types upon differentiation		—		Kalbfuss & Gönczy [126]
*M. musculus (newborn)*	enterocytes at the top of the villus	—	—	—	Komarova & Vorob'ev [127]
*M. musculus*	sperm	—	—	—	Manandhar *et al*. [94]
*R. rattus*	sperm	—	—	—	Woolley & Fawcett [128]
*L. stagnalis*	sperm	—	—	—	Krioutchkova *et al*. [88]
insects (9 species representing Coleoptera, Ephemeroptera, Diptera, Trichoptera, Lepidoptera, Homoptera, and Hemiptera)	sperm	(✓)	—	—	Phillips [129]
many species	oocytes	(✓)			reviewed in Manandhar *et al*. [77]

How prevalent is centriole elimination in a developing organism? This question has been addressed in a comprehensive manner during *C. elegans* embryogenesis [126]. Although worms possess ciliated sensory neurons, they lack motile cilia and flagella. Therefore, the prevalence of centriole elimination can be investigated in the worm without potential confounders stemming from the need to template cilia and flagella. Systematic analysis of animals at the L1 larval stage unveiled that centrioles are eliminated in approximately 88% of cells during *C. elegans* embryogenesis [126]. Detailed lineage analysis revealed that such centriole elimination is stereotyped, occurring at a specific time in each given cell type. Which cells maintain centrioles past embryogenesis? Considering that centrioles are critical for forming centrosomes and thereby directing bipolar spindle assembly in *C. elegans* [132,133], it comes as no surprise that blast cells that proliferate later in development retain centrioles. Moreover, intestinal cells that later undergo endomitoses and endoreduplication cycles also maintain centrioles initially [124,126]. During these endoreduplication cycles, no new centrioles are generated, and the existing ones are eliminated eventually [124] (figure 2*e*). In addition, the systematic analysis revealed that seven terminally differentiated cells in L1 hermaphrodites maintain foci enriched in centriolar proteins, although no ultrastructural analysis was conducted in these cases (box 2). Interestingly, for six of these cells, the analogous cell in the male is a blast cell that later proliferates, suggesting that centriole maintenance in these cases somehow reflects the proliferative potential present in the other sex [126]. Overall, these findings reveal the existence of programmed centriole elimination during *C. elegans* development.

Intriguingly, centriole elimination occurs often in cell types that form syncytia or polyploidize, such as *Drosophila* follicle cells, nurse cells, midgut enterocytes, salivary gland secretory cells, enterocytes and fat cells, or the *C. elegans* intestinal cells mentioned above [113,114,116,117,119,120, 122 124] (table 1). However, multiple examples show that polyploidization or syncitium formation is neither sufficient nor necessary systematically for centriole elimination. Thus, some polyploid tissues maintain centrioles or even amplify them, as exemplified by mammalian polyploid trophoblast giant cells [134]; centrioles are also maintained in other polyploidization instances when cells re-enter mitosis, as in *Drosophila* rectal papillar cells [116]. Furthermore, centriole elimination also occurs in cells in which neither syncytium formation nor polyploidization take place, including most cells during *C. elegans* embryogenesis, as well as ommatidial and interommatidial cells in the *Drosophila* eye [115,126].

**Figure 4.**
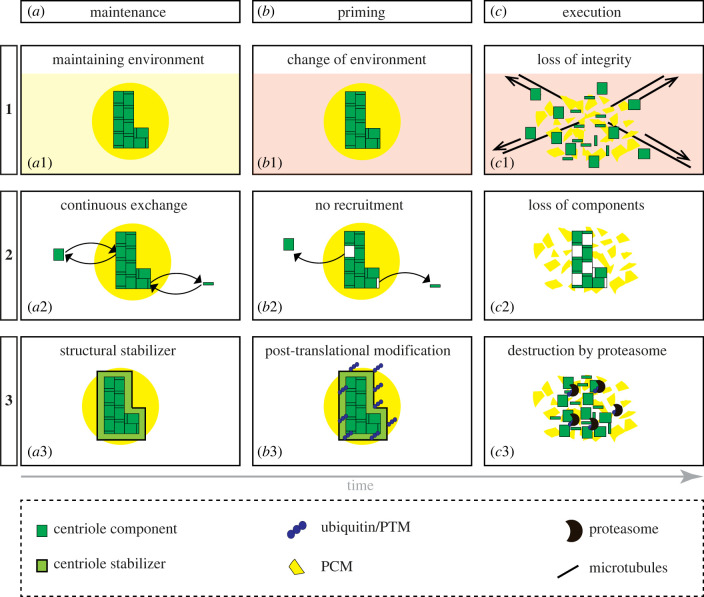
Hypothetical mechanisms underlying stepwise centriole elimination. Three stages that pertain to centriole fate are represented: maintenance (*a*), which concerns stable centrioles, as well as priming (*b*) and execution (*c*), two steps of the elimination process. (*a*) During the centriole maintenance stage, centrioles are exceptionally stable. This could be due to a stabilizing cellular environment (*a*1), to centrioles being continuously repaired through exchange of damaged components with a cytoplasmic pool of intact proteins (*a*2), or to structural stabilizers ensuring centriole integrity (*a*3). (*b*) The priming stage marks the onset of centriole elimination process. This could be due to a change of cellular environment (*b*1), to repair being shut off, for instance because of limited availability of building blocks (*b*2), or to post-translational modifications such as ubiquitination of stabilizer proteins (*b*3). (*c*) In the execution stage, centrioles are destroyed, often together with the surrounding PCM. This could be due to several components being removed at once, perhaps in some cases aided by microtubule-dependent forces pulling on organelle constituents (*c*1). Moreover, the lack of repair could result in irreversible damage of the centriole and thereby to its demise (*c*2). Furthermore, the proteasome might be recruited to poly-ubiquitinated centriolar proteins and remove them from the organelle (*c*3). Note that several of the illustrated mechanisms might occur simultaneously, which would help ensure efficient organelle elimination. Whether all instances of centriole elimination follow the same sequence of events and use the same molecular players is an important question for the future.

The fact that centrioles are eliminated in a stereotyped manner in certain cell types but not others indicates that this process does not occur simply because of inevitable organelle demise with passing time, but instead that elimination is an active process, in which one could distinguish three steps: maintenance, priming and execution (figure 4). Merely exiting the cell cycle is not sufficient for triggering organelle removal, as evidenced by the numerous terminally differentiated cells that harbour cilia or flagella, as well as centrioles. Moreover, in *C. elegans*, some terminally differentiated cells in the adult, including those of the spermatheca, maintain foci enriched in centriolar proteins, whereas others that exited the cell cycle at a later time do not [125]. These observations taken together suggest that centriole elimination can be considered as a manifestation of cell fate. Accordingly, in *C. elegans* embryos, altering the fate of a progenitor that normally yields cells lacking centrioles, such as pharyngeal cells, to that of a progenitor that normally yields cells with centrioles, such as intestinal cells, results in centrioles now being maintained [126]. Likewise, preventing transdifferentiation of a cell that normally maintains centrioles into a cell that normally eliminates them also alters the fate of centrioles, which are now maintained [126].

Overall, the above observations indicate that centriole elimination is by far not restricted to female germ cells and is instead widespread in cells lacking cilia or flagella.

## 6. Why should cells eliminate centrioles?

Why would centrioles need to be eliminated? In the case of oogenesis, the answer is simple: to ensure continuity of the species! Indeed, centriole elimination from the female gamete is essential in most metazoan organisms to ensure correct centriole number in the zygote, thereby leading to bipolar spindle assembly [77,135]. Failure of doing so would put the zygote at risk of assembling a tetrapolar spindle and thereby undergoing chromosome mis-segregation and abortive development. This is exemplified by experiments conducted in *P. miniata*, where experimentally induced retention of mother centrioles in the zygote leads to tetrapolar spindle assembly [87,136–139]. Similarly, in *Drosophila*, maintenance of maternally contributed MTOCs leads to interference with meiotic spindle assembly, mitotic defects and aborted embryonic development [81]. Moreover, in pathological polyspermy, for instance in sea urchins, supernumerary centrioles result in multipolar spindles and chromosome missegregation [140] (reviewed by [141]). Why does centriole elimination occur systematically in female gametes and usually not in male gametes? It is likely that the requirement to have at least one centriole during spermatogenesis to seed axoneme formation of the flagellum has limited the emergence of possible mechanisms during evolution. In principle, however, other modes of centriole inheritance at fertilization that respect this requirement could be envisaged, which may yet be revealed through future work, in particular in non-model organisms. For instance, each gamete could contribute one centriole. Alternatively, each gamete could provide two centrioles without organelle duplication taking place in the first embryonic cell cycle. In addition, it should be noted that centriole elimination during oogenesis can be considered as a barrier against parthenogenesis, as illustrated by experiments in *Xenopus* embryos, where injecting purified human centrosomes can lead to successful parthenogenetic development [142–144].

In contrast to the situation during oogenesis, the putative importance of centriole elimination in terminally differentiated somatic cells remains to be uncovered, although the absence of centrioles in the majority of cells in the worm suggests that centriole maintenance is not innocuous. The current paucity of understanding regarding this putative importance stems in large part from the lack of knowledge regarding the underlying mechanisms, and, thereby, of means to artificially retain centrioles. Consequently, one can only hypothesize about the reasons that may favour centriole elimination from terminally differentiated somatic cells. First, it is possible that centrioles must be removed to ensure that they do not form rogue MTOCs. This may be particularly important in differentiated cell types in which non-centrosomal MTOCs operate (reviewed by [145]). Second, centriole elimination might help prevent inappropriate proliferation. Human cells lacking centrioles arrest at the G1/S transition in a p53-dependent manner [146], such that centriole elimination may provide an extra regulatory step to refrain inappropriate proliferation, and thereby hold tumour suppressive potential. Third, in what is the most hypothetical possibility, perhaps centriole elimination is required to erase information harboured by the organelle, for instance in the form of post-translational modifications (PTMs) of stable constituents. Centrioles are transmitted in a conservative manner across cell generations, endowing their constituents with the potential to carry information over long time spans [147]. Such informational potential is illustrated by the behaviour of stem cells in *Drosophila*, which invariably retain either the centrosome with the older or the younger mother centriole, depending on the tissue [148–151]. Therefore, in essence, temporal information can be encoded in the centriole pedigree and may need to be erased in some circumstances. Finally, centriole elimination might be required in terminally differentiating cells to avoid the formation of primary cilia, which could be dangerous in enabling signalling pathways that rely on this structure.

In summary, in addition to the fundamental importance of oogenesis centriole elimination, future work on organelle elimination mechanisms is expected to enable addressing the importance of this process in specific somatic cells.

## 7. On the temporal and spatial control of centriole elimination

How long does it take to dismantle the centriole organelle? Similar to the variability in the time needed to assemble it, from a few minutes in the early embryos of *Spisula*, *Drosophila* or *C. elegans* [152–154], to several hours in cultured human cells, substantial variability in duration seems to also apply for centriole elimination [155,156]. As mentioned above, ultrastructural analysis of *C. elegans* oogenesis centriole elimination revealed that the initial change in organelle architecture is the loss, in late pachytene, of the central tube, which is located just inside the centriolar microtubule wall [78]. Merely remnants of centriolar microtubules are observed in early diplotene, approximately 4 h thereafter [78]. During *Drosophila* oogenesis, centriolar protein foci diminish in intensity starting at stages 9/12, and are completely undetectable at stage 14 [81], corresponding to approximately one day of development (reviewed by [157]). In the ommatidial and interommatidial cells of the *Drosophila* eye, the centrosomal components γ-tubulin, Cnn and Spd2 disappear at the pupal stages, approximately 25 h after puparium formation (APF). By contrast, Sas4, Cnb, Ana1 and Asl are maintained until the end of pupariation. Ultrastructural analysis uncovered centrioles with slight signs of degeneration at 45–50 h APF, whereas merely centriole remnants are present 60–65 h APF, together suggesting that the elimination process is slow in this case as well [115]. In *C. elegans* embryos, the timing of elimination is cell type specific and more rapid: in certain muscle cells, foci bearing centriolar proteins are absent approximately 95 min after the last mitotic division, whereas such foci are still present greater than 185 min past this time in some sheath cells [126]. Overall, these examples indicate that the timing of centriole elimination can vary substantially depending on the physiologic setting.

Where in the cell do centrioles reside when they disappear? Although occurring often in the vicinity of the nucleus, centriole elimination can also take place elsewhere in the cell. For instance, in *C. elegans*, centriolar foci are located away from the nuclear envelope upon elimination as they migrate through the dendrite of the PQR cell in L1 larvae [158]. In this case, the focus of centriolar SAS-6::GFP disappears when it is approximately 5 µm away from the cell body. Moreover, one of the two centrioles remains close to the nucleus and retains SAS-6 for a longer time [158], raising the possibility that the elimination mechanism is more active further from the nucleus or that the nucleus serves a protective function. Similarly, in the worm embryo, centriole elimination in ciliated neurons begins when these cells start a retrograde migration, with the nucleus moving away from centrioles, which remain at the tip of the dendrite [123]. However, it might be that centriole elimination is initiated in the above cases also when centrioles are still close to the nucleus, but that monitoring merely some centriolar proteins does not reveal this. Compatible with the notion that centrioles are restructured already before their migration, SAS-4 cannot be detected on centrioles of the PQR neuron already when it is still close to the nucleus [158]. Overall, these observations indicate that there might be differences in the sub-cellular location of centrioles being eliminated, depending on the physiological context, but time-resolved ultrastructural analysis will be needed to more precisely determine where in the cell organelle removal initiates.

## 8. On the selectivity of centriole elimination

Are all types of centrioles similarly susceptible to elimination or is there some selectively instead? Female germ cells, for instance in *C. elegans,* possess two pairs of centriole/procentriole following meiotic S phase, which are all subsequently eliminated [159]. Interestingly, the *Drosophila* oocyte inherits many more centrioles than four from the 15 connected nurse cells [113], and somehow manages to eliminate them all. Therefore, centriole elimination in worms and flies can act on both centrioles and procentrioles, and handle more than four organelles in flies. By contrast, the elimination mechanism is daughter-centriole specific in starfish [87]. Indeed, experimentally retaining mother centrioles in the cytoplasm by preventing polar body extrusion results in their maintenance in *P. miniata* [87]. In this experimental setting, mother centrioles maintain a PCM and MTOC activity, whereas both daughter centrioles are eliminated [87]. Therefore, centriole elimination acts specifically on daughter centrioles in this instance. Furthermore, these findings show that the elimination mechanism can handle not just one daughter centriole, as usual, but at least two. Analogous selectivity is observed in another starfish species, *A. forbesi*, except that here the experimentally retained mother centrioles, although persisting, lose MTOC activity [82]. Overall, these observations indicate that, depending on the cellular context, centriole elimination can affect all centrioles present or instead be selective towards a subset of them.

## 9. Targeted centriole elimination during polyspermy

In the case of echinoderms and other species in which centrioles are eliminated during and subsequent to the meiotic divisions, the sperm-derived centrioles are present in the same cytoplasmic milieu as the oocyte-derived daughter centriole, which is about to be eliminated. This begs the question of how sperm-derived centrioles avoid being eliminated in the newly fertilized embryo. In principle, one could imagine that either the elimination mechanism acts locally, or else that paternally and maternally contributed centrioles somehow differ, such that elimination targets solely the organelle stemming from the female gamete.

Selectivity of centriole elimination is even more apparent during physiological polyspermy, where fertilization by multiple sperm cells is required for successful initiation of embryogenesis (reviewed by [160]). In the newt *Cynops pyrrhogaster* and the comb jelly *Beroe ovata*, multiple sperm cells enter the oocyte, but only two large asters of microtubules form, presumably around two sperm-derived centrioles. Therefore, the remaining centrioles must be eliminated or else inactivated [161–163]. Conceivably, the mechanisms leading to removal of sperm nuclei not selected for fusion—called accessory nuclei—might also contribute to elimination of the accompanying centrioles. In *B. ovata,* the female pronucleus migrates towards several sperm-derived nuclei, probing them first, before fusing with only one of them, with the others degenerating thereafter [161,163]. It was proposed that failure of M phase entry leads to degeneration of accessory nuclei, since injection of metaphase-promoting factor (MPF) from unfertilized *Xenopus* eggs into fertilized *Cynops* eggs results in accessory nuclei maintenance and multipolar cleavages, indicative of centrioles being present as well [164]. Normally, accessory nuclei are highly ubiquitinated and enriched in markers of the autophagosome (LC3) and autolysosome (LAMP1), in contrast to the zygote nucleus, suggestive of autophagy contributing to accessory nuclei removal [160]. By extension, perhaps autophagy is involved in eliminating the accompanying centrioles. Furthermore, ubiquitination also primes proteins for degradation via the proteasome (reviewed by [165]), which might also contribute to centriole removal (figure 4*c*3).

How is the choice of aster maintenance upon physiological polyspermy mediated and could the corresponding processes also operate in centriole elimination? Two models that have been suggested for aster maintenance seem particularly interesting to consider in the context of possible analogies with centriole elimination (reviewed by [160]). First, it has been suggested that the choice of which aster is maintained is mediated by differences in the availability of specific proteins, in particular α-/β-/γ-tubulins. By analogy, differences in the cytoplasmic pool of centriolar proteins might be decisive in the balance between centriole maintenance and centriole elimination (figure 4*b*2). Second, it has been suggested that the female pronucleus might be enriched in factors essential for aster maintenance, which would be transported to the closest sperm pronucleus via microtubules. By analogy, perhaps the female pronucleus is also enriched in factors favouring centriole maintenance that are likewise delivered to the salvaged centriole pair.

In summary, physiological polyspermy illustrates in a striking manner that the fate of multiple centrioles can vary within the same cytoplasm.

## 10. How is centriole maintenance achieved?

The fact that centrioles can be eliminated in the first place is particularly remarkable considering the exceptional stability of the organelle in general. Indeed, whereas cytoplasmic microtubules are dynamic and disassemble upon nocodazole or cold treatment, this is not the case for centriolar microtubules, which do not undergo dynamic instability and remain intact under conditions that disassemble cytoplasmic microtubules [147,166]. Furthermore, the axoneme of primary cilia, which is templated by the centriole and similarly bears 9-fold microtubule structures, is dynamic, often disassembling every cell cycle while the centriole is maintained [167]. Similarly, after fertilization, often the sperm axoneme is incorporated into the zygote, but disassembles thereafter, whereas the centrioles remain intact [168]. Moreover, whereas *α*/β-tubulin dimers exhibit high turnover in cytoplasmic microtubules [169,170], they undergo no apparent turnover over one cell cycle in centrioles of human cells [147]. Accordingly, pre-existing centrioles are not eliminated for several days after new centriole formation has been blocked with the Plk4 inhibitor centrinone in p53 negative human cells [171]. Echoing these findings, sperm-derived centrioles marked with β-tubulin::GFP retain fluorescence over several cell cycles in the resulting embryos of *C. elegans* [172]. Likewise, the levels of sperm-contributed centriolar SAS-6::GFP and GFP::SAS-4 remain essentially unchanged, indicating that not only constituent microtubules but also core centriolar proteins are stable once incorporated in the organelle [172].

What confers such striking stability to centrioles, and could an understanding of the underlying maintenance mechanisms help explain how organelle elimination is primed and then executed (figure 4)? First, the presence of triplet and doublet microtubules could be important. Whereas cytoplasmic microtubules are single tube-like polymers typically composed of 13 protofilaments, in most species centriolar microtubules form triplets and doublets with a unique geometry. Triplets consist of one complete microtubule (A-microtubule) containing 13 protofilaments, plus two incomplete ones (B- and C-microtubules), each with 10 protofilaments proper, whereas doublets harbour A- and B-microtubules (figure 1*b*) [105,106,173]. Microtubule triplets are present in the proximal region of the centriole, microtubule doublets in the distal one. The tubulin isoforms δ- and ε-tubulin are needed for triplet and/or doublet formation in *Chlamydomonas reinhardtii* and human cells [3,174,175]. Human cells lacking p53 and δ- or ε-tubulin form centrioles with singlet microtubules, which are unstable and disintegrate during mitosis [3]. The resulting daughter cells initially lack centrioles and then undergo de novo centriole formation. The proteins TEDC1 and TEDC2 may function with δ- and ε-tubulin, with which they associate, given that their deletion phenocopies that of δ- or ε-tubulin [176]. Together, these observations raise the possibility that centriole elimination could begin by removal or weakening of microtubule triplets and doublets. Note, however, that centrioles in *C. elegans* have been reported to harbour singlet microtubules [90] and nevertheless can be extremely stable, as mentioned above, so that stabilization through triplet and doublet microtubules cannot be a universal mechanism.

A second possibility through which exceptional stability could be imparted is via specific stabilizing proteins. In human cells, these include the microtubule-interacting proteins HsPOC1A and HsPOC1B, as well as the PCM component CAP350, although the exact mechanisms through which these proteins exert their stabilizing function are not clear [51,177–180]. In addition, a stabilizing function has been proposed for the daughter-centriole specific component Centrobin [181,182]. Expression of a short fragment of the Centrobin tubulin binding domain (Centrobin-TuBD) leads to centriole loss in approximately 25% of cells. Even though Centrobin is thought to be a procentriole- and daughter centriole-specific component, it was proposed that Centrobin-TuBD, through binding to tubulins, might displace mother centriole proteins that are essential for centriole stability [181]. Furthermore, Centrobin might protect CPAP from degradation by the proteasome (figure 4*a*3–*c*3), as CPAP is absent from centrioles upon Centrobin depletion, a lack that is rescued by proteasome inhibition [183].

Bld10p, the orthologue of vertebrate Cep135 [184], could also serve an important centriole stabilizer function. In *Chlamydomonas*, centrioles do not assemble in the complete absence of Bld10p, but expression of an N-terminally truncated Bld10p construct results in premature cartwheel loss, suggestive of an unstable connection between cartwheel and microtubule triplets [44]. Furthermore, some triplets are missing in this experimental setting. Moreover, *Tetrahymena* cells lacking Bld10p and arrested in G1 to prevent new procentriole formation exhibit centriole number reduction over time [185]. Furthermore, Bld10p stabilizes A- and C-microtubules and helps position triplet microtubules, probably allowing them to withstand forces that act during ciliary beating [185].

Conceivably, changing the turnover rate of stabilizing proteins such as HsPOC1A and HsPOC1B, CEP350, or Cep135/Bld10p might prime centrioles for elimination (figure 4*b*2). A pulsed-SILAC proteomic analysis in human cells uncovered a wide range of turnover rates among 145 centriolar and centrosomal proteins, with an average exchange of approximately 57% of the protein pool over 20 h [186]. NEK2 exhibits the highest turnover, with approximately 96% of the centriolar protein pool exchanged in 20 h, whereas TUBG1 has the lowest, with approximately 22% exchanged in the same period. How dynamic are the components that are known to act as centriolar stabilizers? HsPOC1A and HsPOC1B have turnover rates of approximately 35% and 47%, respectively, Cep350 and Centrobin are more dynamic at approximately 74% and 73%, respectively, whereas Cep135 has an average turnover rate of approximately 56% [186]. Stopping the centriolar incorporation of proteins with high turnover offers the potential to rapidly prime organelle removal (figure 4*a*2–*c*2). By contrast, proteins with low turnover might be primed post-translationally for proteasome-mediated removal (figure 4*a*3–*c*3). However, whether and, if so, how, turnover rates change during centriole elimination has not been investigated.

An additional important stabilizer of centrioles in *C. elegans* is SAS-1, as evidenced by the fact that centrioles derived from *sas-1* mutant sperm lose integrity shortly after fertilization [187,188]. Similarly, if *sas-1* function is lacking maternally, centrioles form initially but disintegrate during embryogenesis. Recently, a role for SAS-1 was also proposed during oogenesis centriole elimination, whereby SAS-1 disappears from centrioles earlier than other components, concomitant with loss of the central tube, to which SAS-1 localizes [78]. Moreover, in *sas-1(t1521ts)* mutant worms, centriolar microtubule and SAS-4 signals decay faster than normal, along with premature loss of organelle integrity [78]. Suggestively, *C. elegans* SAS-1 expressed in human cells associates with and stabilizes microtubules [188]. SAS-1 is related to human C2CD3, which is required to form complete centrioles and the primary cilium in mammals and mice [189–191]. The exact mechanism through which SAS-1, and potentially C2CD3, confers stability to the centriole is unclear. However, given the striking 9-fold radially symmetric distribution of the two proteins uncovered by expansion microscopy, just within the confines of the microtubule wall, as well as their organelle stabilizing function, it is tempting to speculate that these proteins form an inner brace that somehow hold together centriolar microtubules [109,110,192]. Removing this brace may destabilize centrioles, thus potentially offering a handle to modulate organelle elimination.

Finally, centriole integrity might be influenced by PTMs of α- and β-tubulin (figure 4*a*3–*c*3). Centriolar microtubules undergo substantial post-translational modifications, including acetylation, detyrosinylation and polyglutamylation (reviewed by [193]). Injection of antibodies against polyglutamylated tubulin into human cells results in centriole elimination, which has led to the proposal that such PTMs are critical for centriole maintenance [194]. However, since antibody injections in general can lead to degradation of target proteins in a TRIM21-dependent manner [195], it might be that complete removal of centriolar microtubules, and not merely of their PTMs, explains the phenotype observed in the earlier experiments. Further work will be needed to ascertain whether modulating centriolar microtubule PTMs specifically leads to centriole elimination.

In summary, several proteins are known to stabilize centriolar microtubules and to thereby contribute to the striking stability of the organelle, and may offer actionable handles to prime centriole elimination.

## 11. Which factors induce centriole destabilization?

Considering how widespread centriole elimination is, there has been surprisingly little insight to date into the underlying mechanisms, with the important exceptions discussed below. This is especially notable considering that several genome-scale screens have been conducted that might have identified contributing components [189,196,197], as exemplified hereafter in the case of *C. elegans*. Large scale forward genetic and RNAi-based screens deployed in this organism have been instrumental in identifying evolutionarily conserved centriole assembly proteins by imaging early embryos using time-lapse differential interference contrast (DIC) microscopy [187,198–203]. Intriguingly, these screens failed to produce the phenotype that might be expected following failure of oogenesis centriole elimination, namely a tetrapolar spindle during the first division. Several reasons could explain this lack. First, although these screens were extensive, perhaps some components important for oogenesis centriole elimination were not targeted because the genes are small, not predicted, or refractory to RNAi-mediated depletion. Second, genes that act in a redundant manner would likely have been missed. Third, preventing oogenesis centriole elimination may result in an earlier gonadal phenotype, such that genes important for oogenesis centriole elimination are not present among those analysed in the embryo. Fourth, it could be that centrioles contributed by the oocyte upon inactivation of an oogenesis centriole elimination factor would not act as MTOCs, much like the last daughter centriole in the newly fertilized starfish zygote, and thereby escape detection by DIC alone.

Regardless of the reason, candidate screens in the worm have identified the heterochronic protein LIN-41 and the RNA helicase CGH-1 as being somehow involved in timing oogenesis centriole elimination, although compromising them merely delays, and does not abrogate, this process [159,204]. It has been proposed that CGH-1, which is involved in mRNA localization and mRNA stabilization [205], targets an mRNA that encodes a protein promoting elimination [159]. Furthermore, the XX karyotype seems important for centriole elimination during *C. elegans* oogenesis, since a fraction of late prophase I oocytes harbours centrioles in mutant males that possess a female somatic gonad and germ line [159]. However, the molecular nature of the factor(s) modulated by the XX karyotype remains to be uncovered. In the case of centriole elimination in the *C. elegans* intestine, phosphorylation of the PCM protein SPD-2 by PLK-1, transcriptional downregulation of centriole biogenesis genes, as well as proteasome degradation, were proposed to collectively contribute to centriole elimination [124].

One possibility is that priming of centriole elimination merely reflects shutting down of centriole maintenance mechanisms, as evoked above for stabilizing proteins. The PCM was suggested to play an important role in centriole maintenance in this manner, with its removal potentially priming centriole elimination. In *Tetrahymena*, for instance, centrioles become unstable upon depletion of the PCM component γ-tubulin [206], although γ-tubulin's additional localization in the centriole core might be more relevant here [207]. In an analogous manner, joint depletion of the PCM components Asl, D-Plp, Spd2 and Cnn, or that of the Polo kinase, leads to centriole loss in cultured *Drosophila* cells arrested in S phase [81]. Interestingly, during *Drosophila* oogenesis, Polo departs from the PCM prior to PCM removal, whereas expression of Polo fused to the centriolar targeting PACT-domain leads to maintenance of centriolar foci beyond fertilization [81]. These foci act as MTOCs and interact with the spindle, leading to abnormal meiotic divisions; the majority of the resulting embryos arrests in the first mitotic division with scattered DNA and multiple MTOCs [81]. In the absence of EM data, it is unclear whether these supernumerary foci are bona fide centrioles or merely centriolar protein assemblies that serve as MTOCs (box 2). Intriguingly, neither the PCM nor the activity of the Polo-like-kinase Plk1 seem sufficient to protect centrioles in other systems. Thus, mother centrioles in *A. forbesi* persist after their experimental retention in the oocyte, although they do not nucleate microtubules [82]. Furthermore, pharmacological inhibition of Plk1 does not lead to premature elimination of centrioles in *P. miniata* [82]. Likewise, in *C. elegans*, centrioles are eliminated from the ciliary base despite the presence of PCM components [130,131]. Moreover, PLK-1 is absent from centriolar foci in the L1 larvae and from the germline, except in the mitotic zone, whereas depletion of PLK-1, PLK-2 and PLK-3 does not lead to precocious elimination during oogenesis [78,125,208]. Regardless, it will be interesting to discover what governs Polo removal from centrioles in *Drosophila*, and investigate the consequences of such removal. Ana1, which localizes to the centriolar wall [46], might be important in this context and act downstream of Polo-mediated removal [209]. Indeed, Ana1 depletion from S-phase arrested *Drosophila* cells promotes centriole loss, whereas expression of Polo-PACT in cells lacking Ana1 does not result in supernumerary centriolar foci, together suggesting that Ana1 needs to be constantly replenished at the centriole by exchange with the cytoplasmic protein pool [209].

Overall, whereas Polo and PCM removal are critical for centriole elimination in *Drosophila*, including during oogenesis, these mechanisms do not appear to be systematically used in other systems for priming and executing centriole elimination.

## 12. Perspectives

In conclusion, we reviewed how the last decades have uncovered aspects of centriole elimination and led to the identification of candidate proteins that might modulate this process. Although the underlying mechanisms still need to be elucidated in most instances, the field already possesses a broad understanding regarding when and where centrioles are eliminated, and has increasingly numerous tools in hand to further tackle this problem.

To shed more light on the mechanisms governing centriole elimination, screens specifically designed to target this process will be instrumental. Genome-scale mutant, RNAi or CRISPR/Cas9-screens aimed at identifying conditions with abrogated or accelerated centriole elimination, not only during oogenesis but also in somatic cells, are expected to identify novel candidates of importance. Given that centriole architecture and assembly mechanisms are widely conserved across species, and since elimination is widespread across the eukaryotic tree of life, it is likely that lessons can be learned about general mechanisms from advances in diverse systems. Emerging model organisms might be helpful in this respect. One such system that may prove particularly useful is the excavate *Naegleria gruberi,* which can transform rapidly from an amoeboid form lacking centrioles to a flagellate form with two centrioles [111,112]. Whereas *Naegleria* has been used to analyse de novo centriole formation during this transformation (reviewed by [210]), it could conceivably serve also to study centriole elimination during the flagellate to amoeboid conversion. Furthermore, lessons might also be learned from other biological systems that can similarly shift from a stable state to an unstable one, such as viral capsids (box 3).

Box 3. **Learning from viral uncoating?**

The origin of centrioles is unclear (reviewed by [211–213]). Considering the extreme ultrastructural similarity of the organelle when comparing species across branches of the eukaryotic tree of life, as well the presence of shared core centriolar proteins throughout these species, the centriole organelle must have been present in the last eukaryotic common ancestor (LECA), over two billion years ago. Given that there is no apparent trace of current centriolar proteins in the extant genomes of prokaryotes or archaea, one possibility is that centrioles emanated from viruses that are long extinct. The dimensions of the centriole and the self-assembly mode of organelle biogenesis are certainly compatible with this hypothesis. While we might never know for certain whether the centriole had a viral origin, regardless, it may be interesting to consider mechanisms governing virus disassembly to entertain possible parallels that may operate during centriole elimination.

Viruses assemble a stable capsid that sustains the harsh extracellular environment but needs to disassemble within cells after infection. Viruses use diverse strategies of viral capsid uncoating to ensure release of their genetic content into the host cell (reviewed by [214]). Hereafter, we discuss some of these mechanisms using adenoviruses as an example, and suggest possible parallels for centriole elimination.

Adenoviruses are non-enveloped viruses that escape endosomes and therefore localize in the capsid form in the cytoplasm, before uncoating in the vicinity of the nuclear envelope [215] (reviewed by [216]). How do adenoviruses shift from a maintenance mode to a disassembly mode of their proteinaceous capsid, and could analogous mechanisms operate for centrioles? First, the environment in which capsids assemble differs from that in which the capsid disassembles. Such a change in environment is conferred by the lower pH encountered by the virus in the endosome, which can lead to conformational changes of viral capsid proteins and therefore promote uncoating (reviewed by [214]). Likewise, during centriole elimination, perhaps changes in the local cellular environment favour disassembly of centriolar proteins (figure 4*b*1,*c*1). Potentially illustrating such changes, centriole elimination occurs during oocyte maturation and terminal differentiation, which are both accompanied by substantial restructuring of the cellular environment.

Adenoviruses also use cytoskeletal-based force mechanisms to ensure efficient capsid disassembly. Thus, adenoviruses dock on the nuclear pore through an interaction with the nucleoporin Nup214 and attach to the microtubule-dependent motor protein kinesin-1 [215]. The forces generated by kinesin-1 then lead to fragmentation of nucleoporins and the attached capsid, the constituents of which are then released in the cytoplasm [215]. Intriguingly, human cells with centrioles made solely of singlet microtubules following δ-/*ε* tubulin deletion fall apart in a microtubule-dependent manner during mitosis [3]. By extension, cytoskeletal-based force mechanisms might contribute to the execution of centriole elimination (figure 4*c*1).

Adenoviruses also employ the ubiquitination and proteasome machineries to help dismantle their capsid, as illustrated by the fact that the host factor E3 ubiquitin ligase Mib1 is essential for uncoating of adenovirus at the nuclear pore [217]. By analogy, proteins that normally stabilize centrioles might be ubiquitinated and then destroyed by the proteasome in the case of centriole elimination (figure 4*c*3).

Finally, we might also learn more about centriole elimination mechanisms by understanding how viruses hijack these mechanisms. For instance, after vaccinia virus infection of HeLa cells, microtubule organization is restructured and centrosomal components are absent [218]. It has not been reported whether centrioles are also eliminated in this case, but this seems plausible given the phenotype.

In summary, viruses employ numerous mechanisms to disassemble their proteinaceous capsids, as exemplified above with adenoviruses. Given the apparent resemblance between viruses and centrioles, and perhaps their shared origin, it might be that the centriole organelle employs similar mechanisms to those known in viruses to shift the equilibrium from assembling and maintaining a stable structure to eliminating it.

There is clear evidence that centriole elimination is widespread and directed by cell fate. A process with similar breadth and regulation is programmed cell death or apoptosis (reviewed by [219,220]). Just as apoptosis proved critical in many physiological conditions, including development, homeostasis, and immune function, centriole elimination might play a role in more processes than originally anticipated from the pioneering work of Boveri. Decades of work elucidating the mechanisms governing apoptosis have uncovered complex pathways that can be finely regulated in a manner appropriate for each physiological setting. Looking ahead, we anticipate that the same will hold for centriole elimination.

Just like apoptosis gone awry can contribute to numerous pathological conditions (reviewed by [219,220]), inappropriate centriole elimination may hold important implications for disease. For instance, defective centriole elimination might lead to female sterility: in principle, failing to eliminate centrioles from the oocyte could lead to multipolar spindle assembly in the first division of the zygote and developmental arrest. By contrast, precocious centriole elimination might result in male sterility, as well as abnormal embryonic development owing to defective cell polarity, signalling and division. It will be interesting also to investigate whether faulty centriole elimination mechanisms contribute to tumorigenesis. Experimentally induced centriole amplification is sufficient to drive aneuploidy and subsequent tumorigenesis in multiple mouse tissues [221–223]. It appears reasonable to think that lowering centriole number may also impact tumorigenesis. Mitotic errors, including lagging chromatids, micronuclei, aneuploidy and polyploidy, are widespread in proliferating cells lacking centrioles and p53 [171,224–226]. Moreover, cells lacking centrioles have been suggested to drive genomic instability in early primary prostate tumours [227].

Overall, the contribution of centriole elimination in disease settings is under-explored but might be important for both understanding underlying mechanisms and envisaging novel therapeutic avenues.

The path ahead is exciting—the field is on the brink of understanding a very fundamental process in cell and developmental biology.

## Data Availability

No new data were generated in this review.

## References

[1] Boveri T. 1887 Ueber den Antheil des Spermatozoon an der Theilung des Eies. Sitzungsberichte der Gesellschaft für Morphologie und Physiologie in München 3. Munich, Germany.

[2] Chang P, Stearns T. 2000 δ-Tubulin and ɛ-tubulin: two new human centrosomal tubulins reveal new aspects of centrosome structure and function. Nat. Cell Biol. **2**, 30-35. (10.1038/71350) 10620804

[3] Wang JT, Kong D, Hoerner CR, Loncarek J, Stearns T. 2017 Centriole triplet microtubules are required for stable centriole formation and inheritance in human cells. ELife **6**, e29061. (10.7554/eLife.29061) 28906251 PMC5653238

[4] Gupta A, Kitagawa D. 2018 Ultrastructural diversity between centrioles of eukaryotes. J. Biochem. **164**, 1-8. (10.1093/jb/mvy031) 29462371

[5] Paintrand M, Moudjou M, Delacroix H, Bornens M. 1992 Centrosome organization and centriole architecture: Their sensitivity to divalent cations. J. Struct. Biol. **108**, 107-128. (10.1016/1047-8477(92)90011-X) 1486002

[6] Hirono M. 2014 Cartwheel assembly. Phil. Trans. R. Soc. B **369**, 20130458. (10.1098/rstb.2013.0458) 25047612 PMC4113102

[7] Bornens M. 2012 The centrosome in cells and organisms. Science **335**, 422-426. (10.1126/science.1209037) 22282802

[8] Gönczy P. 2012 Towards a molecular architecture of centriole assembly. Nat. Rev. Mol. Cell Biol. **13**, 425-435. (10.1038/nrm3373) 22691849

[9] LeGuennec M, Klena N, Aeschlimann G, Hamel V, Guichard P. 2021 Overview of the centriole architecture. Curr. Opin. Struct. Biol. **66**, 58-65. (10.1016/j.sbi.2020.09.015) 33176264

[10] Winey M, O'Toole E. 2014 Centriole structure. Phil. Trans. R. Soc. B **369**, 20130457. (10.1098/rstb.2013.0457) 25047611 PMC4113101

[11] Breslow DK, Holland AJ. 2019 Mechanism and regulation of centriole and cilium biogenesis. Annu. Rev. Biochem. **88**, 691-724. (10.1146/annurev-biochem-013118-111153) 30601682 PMC6588485

[12] Pintard L, Bowerman B. 2019 Mitotic Cell Division in Caenorhabditis elegans. Genetics **211**, 35-73. (10.1534/genetics.118.301367) 30626640 PMC6325691

[13] Bettencourt-Dias M, Hildebrandt F, Pellman D, Woods G, Godinho SA. 2011 Centrosomes and cilia in human disease. Trends Genet. **27**, 307-315. (10.1016/j.tig.2011.05.004) 21680046 PMC3144269

[14] Braun DA, Hildebrandt F. 2017 Ciliopathies. Cold Spring Harb. Perspect. Biol. **9**, a028191. (10.1101/cshperspect.a028191) 27793968 PMC5334254

[15] Klena NT, Gibbs BC, Lo CW. 2017 Cilia and ciliopathies in congenital heart disease. Cold Spring Harb. Perspect. Biol. **9**, 8. (10.1101/cshperspect.a028266) PMC5538412 28159874

[16] Banterle N, Gönczy P. 2017 Centriole biogenesis: from identifying the characters to understanding the plot. Annu. Rev. Cell Dev. Biol. **33**, 23-49. (10.1146/annurev-cellbio-100616-060454) 28813178

[17] Brito DA, Gouveia SM, Bettencourt-Dias M. 2012 Deconstructing the centriole: structure and number control. Curr. Opin. Cell Biol. **24**, 4-13. (10.1016/j.ceb.2012.01.003) 22321829

[18] Gomes Pereira S, Dias Louro MA, Bettencourt-Dias M. 2021 Biophysical and quantitative principles of centrosome biogenesis and structure. Annu. Rev. Cell Dev. Biol. **37**, 43-63. (10.1146/annurev-cellbio-120219-051400) 34314592

[19] Nigg EA, Holland AJ. 2018 Once and only once: mechanisms of centriole duplication and their deregulation in disease. Nat. Rev. Mol. Cell Biol. **19**, 297-312. (10.1038/nrm.2017.127) 29363672 PMC5969912

[20] Fujita H, Yoshino Y, Chiba N. 2016 Regulation of the centrosome cycle. Mol. Cell. Oncol. **3**, e1075643. (10.1080/23723556.2015.1075643) 27308597 PMC4905396

[21] Kong D, Farmer V, Shukla A, James J, Gruskin R, Kiriyama S, Loncarek J. 2014 Centriole maturation requires regulated Plk1 activity during two consecutive cell cycles. J. Cell Biol. **206**, 855-865. (10.1083/jcb.201407087) 25246616 PMC4178969

[22] Cizmecioglu O, Arnold M, Bahtz R, Settele F, Ehret L, Haselmann-Weiß U, Antony C, Hoffmann I. 2010 Cep152 acts as a scaffold for recruitment of Plk4 and CPAP to the centrosome. J. Cell Biol. **191**, 731-739. (10.1083/jcb.201007107) 21059844 PMC2983070

[23] Gomez-Ferreria MA, Rath U, Buster DW, Chanda SK, Caldwell JS, Rines DR, Sharp DJ. 2007 Human Cep192 is required for mitotic centrosome and spindle assembly. Curr. Biol. **17**, 1960-1966. (10.1016/j.cub.2007.10.019) 17980596

[24] Hatch EM, Kulukian A, Holland AJ, Cleveland DW, Stearns T. 2010 Cep152 interacts with Plk4 and is required for centriole duplication. J. Cell Biol. **191**, 721-729. (10.1083/jcb.201006049) 21059850 PMC2983069

[25] Joukov V, Nicolo AD, Rodriguez A, Walter JC, Livingston DM. 2010 Centrosomal protein of 192 kDa (Cep192) promotes centrosome-driven spindle assembly by engaging in organelle-specific Aurora A activation. Proc. Natl Acad. Sci. USA **107**, 21 022-21 027. (10.1073/pnas.1014664107) PMC3000277 21097701

[26] Lukinavičius G, Lavogina D, Orpinell M, Umezawa K, Reymond L, Garin N, Gönczy P, Johnsson K. 2013 Selective chemical crosslinking reveals a Cep57-Cep63-Cep152 centrosomal complex. Curr. Biol. **23**, 265-270. (10.1016/j.cub.2012.12.030) 23333316

[27] Sir J-H et al. 2011 A primary microcephaly protein complex forms a ring around parental centrioles. Nat. Genet. **43**, 1147-1153. (10.1038/ng.971) 21983783 PMC3299569

[28] Sonnen KF, Schermelleh L, Leonhardt H, Nigg EA. 2012 3D-structured illumination microscopy provides novel insight into architecture of human centrosomes. Biol. Open **1**, 965-976. (10.1242/bio.20122337) 23213374 PMC3507176

[29] Zhu F et al. 2008 The mammalian SPD-2 ortholog Cep192 regulates centrosome biogenesis. Curr. Biol. **18**, 136-141. (10.1016/j.cub.2007.12.055) 18207742

[30] Brownlee CW, Klebba JE, Buster DW, Rogers GC. 2011 The protein phosphatase 2A regulatory subunit twins stabilizes Plk4 to induce centriole amplification. J. Cell Biol. **195**, 231-243. (10.1083/jcb.201107086) 21987638 PMC3198173

[31] Cunha-Ferreira I, Rodrigues-Martins A, Bento I, Riparbelli M, Zhang W, Laue E, Callaini G, Glover DM, Bettencourt-Dias M. 2009 The SCF/Slimb ubiquitin ligase limits centrosome amplification through degradation of SAK/PLK4. Curr. Biol. **19**, 43-49. (10.1016/j.cub.2008.11.037) 19084407

[32] Guderian G, Westendorf J, Uldschmid A, Nigg EA. 2010 Plk4 trans -autophosphorylation regulates centriole number by controlling *β*TrCP-mediated degradation. J. Cell Sci. **123**, 2163-2169. (10.1242/jcs.068502) 20516151

[33] Holland AJ, Lan W, Niessen S, Hoover H, Cleveland DW. 2010 Polo-like kinase 4 kinase activity limits centrosome overduplication by autoregulating its own stability. J. Cell Biol. **188**, 191-198. (10.1083/jcb.200911102) 20100909 PMC2813471

[34] Rogers GC, Rusan NM, Roberts DM, Peifer M, Rogers SL. 2009 The SCFSlimb ubiquitin ligase regulates Plk4/Sak levels to block centriole reduplication. J. Cell Biol. **184**, 225-239. (10.1083/jcb.200808049) 19171756 PMC2654306

[35] Sillibourne JE, Tack F, Vloemans N, Boeckx A, Thambirajah S, Bonnet P, Ramaekers FCS, Bornens M, Grand-Perret T. 2010 Autophosphorylation of Polo-like kinase 4 and its role in centriole duplication. Mol. Biol. Cell **21**, 547-561. (10.1091/mbc.e09-06-0505) 20032307 PMC2820420

[36] Sillibourne JE, Specht CG, Izeddin I, Hurbain I, Tran P, Triller A, Darzacq X, Dahan M, Bornens M. 2011 Assessing the localization of centrosomal proteins by PALM/STORM nanoscopy. Cytoskeleton **68**, 619-627. (10.1002/cm.20536) 21976302

[37] Arquint C, Gabryjonczyk A-M, Imseng S, Böhm R, Sauer E, Hiller S, Nigg EA, Maier T. 2015 STIL binding to Polo-box 3 of PLK4 regulates centriole duplication. ELife **4**, e07888. (10.7554/eLife.07888) 26188084 PMC4530586

[38] Kratz A-S, Bärenz F, Richter KT, Hoffmann I. 2015 Plk4-dependent phosphorylation of STIL is required for centriole duplication. Biol. Open **4**, 370-377. (10.1242/bio.201411023) 25701666 PMC4359743

[39] Moyer TC, Clutario KM, Lambrus BG, Daggubati V, Holland AJ. 2015 Binding of STIL to Plk4 activates kinase activity to promote centriole assembly. J. Cell Biol. **209**, 863-878. (10.1083/jcb.201502088) 26101219 PMC4477857

[40] Ohta M, Ashikawa T, Nozaki Y, Kozuka-Hata H, Goto H, Inagaki M, Oyama M, Kitagawa D. 2014 Direct interaction of Plk4 with STIL ensures formation of a single procentriole per parental centriole. Nat. Commun. **5**, 5267. (10.1038/ncomms6267) 25342035 PMC4220463

[41] Kitagawa D et al. 2011 Structural basis of the 9-fold symmetry of centrioles. Cell **144**, 364-375. (10.1016/j.cell.2011.01.008) 21277013 PMC3089914

[42] van Breugel M et al. 2011 Structures of SAS-6 suggest its organization in centrioles. Science **331**, 1196-1199. (10.1126/science.1199325) 21273447

[43] Carvalho-Santos Z et al. 2012 BLD10/CEP135 is a microtubule-associated protein that controls the formation of the flagellum central microtubule pair. Dev. Cell **23**, 412-424. (10.1016/j.devcel.2012.06.001) 22898782

[44] Hiraki M, Nakazawa Y, Kamiya R, Hirono M. 2007 Bld10p constitutes the cartwheel-spoke tip and stabilizes the 9-fold symmetry of the centriole. Curr. Biol. **17**, 1778-1783. (10.1016/j.cub.2007.09.021) 17900905

[45] Kraatz S et al. 2016 The human centriolar protein CEP135 contains a two-stranded coiled-coil domain critical for microtubule binding. Structure **24**, 1358-1371. (10.1016/j.str.2016.06.011) 27477386

[46] Tian Y, Wei C, He J, Yan Y, Pang N, Fang X, Liang X, Fu J. 2021 Superresolution characterization of core centriole architecture. J. Cell Biol. **220**, 4. (10.1083/jcb.202005103) PMC7863704 33533934

[47] Hatzopoulos GN, Erat MC, Cutts E, Rogala KB, Slater LM, Stansfeld PJ, Vakonakis I. 2013 Structural analysis of the G-box domain of the microcephaly protein CPAP suggests a role in centriole architecture. Structure **21**, 2069-2077. (10.1016/j.str.2013.08.019) 24076405 PMC3824074

[48] Moyer TC, Holland AJ. 2019 PLK4 promotes centriole duplication by phosphorylating STIL to link the procentriole cartwheel to the microtubule wall. ELife **8**, e46054. (10.7554/eLife.46054) 31115335 PMC6570480

[49] Tang C-JC, Lin S-Y, Hsu W-B, Lin Y-N, Wu C-T, Lin Y-C, Chang C-W, Wu K-S, Tang TK. 2011 The human microcephaly protein STIL interacts with CPAP and is required for procentriole formation. EMBO J. **30**, 4790-4804. (10.1038/emboj.2011.378) 22020124 PMC3243611

[50] Comartin D et al. 2013 CEP120 and SPICE1 cooperate with CPAP in centriole elongation. Curr. Biol. **23**, 1360-1366. (10.1016/j.cub.2013.06.002) 23810536

[51] Keller LC, Geimer S, Romijn E, Yates J, Zamora I, Marshall WF. 2009 Molecular architecture of the centriole proteome: the conserved WD40 domain protein POC1 is required for centriole duplication and length control. Mol. Biol. Cell **20**, 1150-1166. (10.1091/mbc.e08-06-0619) 19109428 PMC2642750

[52] Kohlmaier G, Lončarek J, Meng X, McEwen BF, Mogensen MM, Spektor A, Dynlacht BD, Khodjakov A, Gönczy P. 2009 Overly long centrioles and defective cell division upon excess of the SAS-4-related protein CPAP. Curr. Biol. **19**, 1012-1018. (10.1016/j.cub.2009.05.018) 19481460 PMC2993638

[53] Schmidt TI, Kleylein-Sohn J, Westendorf J, Clech ML, Lavoie SB, Stierhof Y-D, Nigg EA. 2009 Control of centriole length by CPAP and CP110. Curr. Biol. **19**, 1005-1011. (10.1016/j.cub.2009.05.016) 19481458

[54] Spektor A, Tsang WY, Khoo D, Dynlacht BD. 2007 Cep97 and CP110 suppress a cilia assembly program. Cell **130**, 678-690. (10.1016/j.cell.2007.06.027) 17719545

[55] Tang C-JC, Fu R-H, Wu K-S, Hsu W-B, Tang TK. 2009 CPAP is a cell-cycle regulated protein that controls centriole length. Nat. Cell Biol. **11**, 825-831. (10.1038/ncb1889) 19503075

[56] Laoukili J, Perret E, Middendorp S, Houcine O, Guennou C, Marano F, Bornens M, Tournier F. 2000 Differential expression and cellular distribution of centrin isoforms during human ciliated cell differentiation in vitro. J. Cell Sci. **113**, 1355-1364. (10.1242/jcs.113.8.1355) 10725219

[57] Le Guennec M et al. 2020 A helical inner scaffold provides a structural basis for centriole cohesion. Sci. Adv. **6**, eaaz4137. (10.1126/sciadv.aaz4137) 32110738 PMC7021493

[58] Middendorp S, Küntziger T, Abraham Y, Holmes S, Bordes N, Paintrand M, Paoletti A, Bornens M. 2000 A role for centrin 3 in centrosome reproduction. J. Cell Biol. **148**, 405-416. (10.1083/jcb.148.3.405) 10662768 PMC2174797

[59] Paoletti A, Moudjou M, Paintrand M, Salisbury JL, Bornens M. 1996 Most of centrin in animal cells is not centrosome-associated and centrosomal centrin is confined to the distal lumen of centrioles. J. Cell Sci. **109**, 3089-3102. (10.1242/jcs.109.13.3089) 9004043

[60] Steib E et al. 2020 WDR90 is a centriolar microtubule wall protein important for centriole architecture integrity. ELife **9**, e57205. (10.7554/eLife.57205) 32946374 PMC7500955

[61] Takumi K, Kitagawa D. 2022 Experimental and natural induction of de novo centriole formation. Front. Cell Dev. Biol. **10**, 861864. (10.3389/fcell.2022.861864) 35445021 PMC9014216

[62] Courtois A, Schuh M, Ellenberg J, Hiiragi T. 2012 The transition from meiotic to mitotic spindle assembly is gradual during early mammalian development. J. Cell Biol. **198**, 357-370. (10.1083/jcb.201202135) 22851319 PMC3413348

[63] Gomes Pereira S et al. 2021 The 3D architecture and molecular foundations of de novo centriole assembly via bicentrioles. Curr. Biol. **31**, 4340-4353.e7. (10.1016/j.cub.2021.07.063) 34433076

[64] Gueth-Hallonet C, Antony C, Aghion J, Santa-Maria A, Lajoie-Mazenc I, Wright M, Maro B. 1993 γ-Tubulin is present in acentriolar MTOCs during early mouse development. J. Cell Sci. **105**, 157-166. (10.1242/jcs.105.1.157) 8360270

[65] Khodjakov A, Rieder CL, Sluder G, Cassels G, Sibon O, Wang C-L. 2002 De novo formation of centrosomes in vertebrate cells arrested during S phase. J. Cell Biol. **158**, 1171-1181. (10.1083/jcb.200205102) 12356862 PMC2173237

[66] Wang W-J, Acehan D, Kao C-H, Jane W-N, Uryu K, Tsou M-FB. 2015 De novo centriole formation in human cells is error-prone and does not require SAS-6 self-assembly. ELife **4**, e10586. (10.7554/eLife.10586) 26609813 PMC4709270

[67] Dirksen ER. 1971 Centriole morphogenesis in developing ciliated epithelium of the mouse oviduct. J. Cell Biol. **51**, 286-302. (10.1083/jcb.51.1.286) 5111878 PMC2108250

[68] Kalnins VI, Porter KR. 1969 Centriole replication during ciliogenesis in the chick tracheal epithelium. Zeitschrift Für Zellforschung Und Mikroskopische Anatomie **100**, 1-30. (10.1007/BF00343818) 5354183

[69] Sorokin SP. 1968 Reconstructions of centriole formation and ciliogenesis in mammalian lungs. J. Cell Sci. **3**, 207-230. (10.1242/jcs.3.2.207) 5661997

[70] Steinman RM. 1968 An electron microscopic study of ciliogenesis in developing epidermis and trachea in the embryo of *Xenopus laevis*. Am. J. Anat. **122**, 19-55. (10.1002/aja.1001220103) 5654501

[71] Bernhard W, Harven ED. 1956 Sur la présence dans certaines cellules de mammifères d'un organite de nature probablement centriolaire: étude au microscope électronique. Comptes Rendus Hebdomadaires Des Seances de l'Academie Des Sciences **242**, 288-290. 13305018

[72] Harven ED, Bernhard W. 1956 Etude au microscope electronique de l'ultrastructure du centriole chez les vertébrés. Zeitschrift Für Zellforschung Und Mikroskopische Anatomie **45**, 378-398. (10.1007/BF01106086) 13402087

[73] Hertig AT, Adams EC. 1967 Studies on the human oocyte and its follicle. J. Cell Biol. **34**, 647-675. (10.1083/jcb.34.2.647) 4292010 PMC2107315

[74] Zamboni L, Mastroianni L. 1966 Electron microscopic studies on rabbit ova. J. Ultrastruct. Res. **14**, 95-117. (10.1016/S0022-5320(66)80038-2)

[75] Januschke J, Gervais L, Gillet L, Keryer G, Bornens M, Guichet A. 2006 The centrosome-nucleus complex and microtubule organization in the Drosophila oocyte. Development **133**, 129-139. (10.1242/dev.02179) 16319114

[76] Szollosi D, Calarco P, Donahue RP. 1972 Absence of centrioles in the first and second meiotic spindles of mouse oocytes. J. Cell Sci. **11**, 521-541. (10.1242/jcs.11.2.521) 5076360

[77] Manandhar G, Schatten H, Sutovsky P. 2005 Centrosome reduction during gametogenesis and its significance. Biol. Reprod. **72**, 2-13. (10.1095/biolreprod.104.031245) 15385423

[78] Pierron M, Woglar A, Busso C, Jha K, Mikeladze-Dvali T, Croisier M, Gönczy P. 2023 Centriole elimination during *C. elegans* oogenesis initiates with loss of the central tube protein SAS-1. *BioRxiv*. (10.1101/2023.06.19.545600) PMC10711648 37987153

[79] Becalska AN, Gavis ER. 2009 Lighting up mRNA localization in Drosophila oogenesis. Development **136**, 2493-2503. (10.1242/dev.032391) 19592573 PMC2709059

[80] Gonzalez C, Tavosanis G, Mollinari C. 1998 Centrosomes and microtubule organisation during Drosophila development. J. Cell Sci. **111**, 2697-2706. (10.1242/jcs.111.18.2697) 9718363

[81] Pimenta-Marques A, Bento I, Lopes CAM, Duarte P, Jana SC, Bettencourt-Dias M. 2016 A mechanism for the elimination of the female gamete centrosome in Drosophila melanogaster. Science **353**, aaf4866. (10.1126/science.aaf4866) 27229142

[82] Pierron M, Kalbfuss N, Borrego-Pinto J, Lénárt P, Gönczy P. 2020 Centriole foci persist in starfish oocytes despite Polo-like kinase 1 inactivation or loss of microtubule nucleation activity. Mol. Biol. Cell **31**, 873-880. (10.1091/mbc.E19-06-0346) 32073992 PMC7185973

[83] Kato KH, Washitani-Nemoto S, Hino A, Nemoto S. 1990 Ultrastructural Studies on the Behavior of Centrioles during Meiosis of Starfish Oocytes. Development, Growth and Differentiation **32**, 41-49. (10.1111/j.1440-169X.1990.00041.x) 37281512

[84] Longo FJ, Anderson E. 1969 Sperm differentiation in the sea urchins *Arbacia punctulata* and *Strongylocentrotus purpuratus*. J. Ultrastruct. Res. **27**, 486-509. (10.1016/S0022-5320(69)80046-8) 5816822

[85] Miyazaki A, Kato KH, Nemoto S. 2005 Role of microtubules and centrosomes in the eccentric relocation of the germinal vesicle upon meiosis reinitiation in sea-cucumber oocytes. Dev. Biol. **280**, 237-247. (10.1016/j.ydbio.2005.01.026) 15766762

[86] Nakashima S, Kato KH. 2001 Centriole behavior during meiosis in oocytes of the sea urchin *Hemicentrotus pulcherrimus*. Dev. Growth Differ. **43**, 437-445. (10.1046/j.1440-169x.2001.00580.x) 11473550

[87] Borrego-Pinto J, Somogyi K, Karreman MA, König J, Müller-Reichert T, Bettencourt-Dias M, Gönczy P, Schwab Y, Lénárt P. 2016 Distinct mechanisms eliminate mother and daughter centrioles in meiosis of starfish oocytes. J. Cell Biol. **212**, 815-827. (10.1083/jcb.201510083) 27002173 PMC4810307

[88] Krioutchkova MM, Onishchenko GE, Chentsov YS. 1994 An Ultrastructural study of the centrosome and centrioles in gametogenesis and early embryogenesis of *Lymnaea stagnalis*. J. Struct. Biol. **112**, 59-69. (10.1006/jsbi.1994.1007)

[89] Komaru A, Ookubo K, Kiyomoto M. 2000 All meiotic chromosomes and both centrosomes at spindle pole in the zygotes discarded as two polar bodies in clam *Corbicula leana*: unusual polar body formation observed by antitubulin immunofluorescence. Dev. Genes Evol. **210**, 263-269. (10.1007/s004270050313) 11180831

[90] Wolf N, Hirsh D, McIntosh JR. 1978 Spermatogenesis in males of the free-living nematode, Caenorhabditis elegans. J. Ultrastruct. Res. **63**, 155-169. (10.1016/S0022-5320(78)80071-9) 671581

[91] Kuriyama R, Kanatani H. 1981 The centriolar complex isolated from starfish spermatozoa. J. Cell Sci. **49**, 33-49. (10.1242/jcs.49.1.33) 7031073

[92] Albertson DG. 1984 Formation of the first cleavage spindle in nematode embryos. Dev. Biol. **101**, 61-72. (10.1016/0012-1606(84)90117-9) 6692980

[93] Kirkham M, Müller-Reichert T, Oegema K, Grill S, Hyman AA. 2003 SAS-4 is a C. elegans centriolar protein that controls centrosome size. Cell **112**, 575-587. (10.1016/S0092-8674(03)00117-X) 12600319

[94] Manandhar G, Simerly C, Schatten G. 1999 Centrosome reduction during mammalian spermiogenesis. Curr. Topics Dev. Biol. **49**, 343-363. (10.1016/S0070-2153(99)49017-9) 11005027

[95] Avidor-Reiss T, Carr A, Fishman EL. 2020 The sperm centrioles. Mol. Cell. Endocrinol. **518**, 110987. (10.1016/j.mce.2020.110987) 32810575 PMC7606549

[96] Fishman EL et al. 2018 A novel atypical sperm centriole is functional during human fertilization. Nat. Commun. **9**, 2210. (10.1038/s41467-018-04678-8) 29880810 PMC5992222

[97] Blachon S, Cai X, Roberts KA, Yang K, Polyanovsky A, Church A, Avidor-Reiss T. 2009 A proximal centriole-like structure is present in Drosophila spermatids and can serve as a model to study centriole duplication. Genetics **182**, 133-144. (10.1534/genetics.109.101709) 19293139 PMC2674812

[98] Blachon S, Khire A, Avidor-Reiss T. 2014 The origin of the second centriole in the zygote of *Drosophila melanogaster*. Genetics **197**, 199-205. (10.1534/genetics.113.160523) 24532732 PMC4012480

[99] Khire A et al. 2016 Centriole remodeling during spermiogenesis in Drosophila. Curr. Biol. **26**, 3183-3189. (10.1016/j.cub.2016.07.006) 28094036 PMC5245371

[100] Khire A, Vizuet AA, Davila E, Avidor-Reiss T. 2015 Asterless reduction during spermiogenesis is regulated by Plk4 and is essential for zygote development in Drosophila. Curr. Biol. **25**, 2956-2963. (10.1016/j.cub.2015.09.045) 26480844 PMC4654664

[101] Kai Y, Iwata K, Iba Y, Mio Y. 2015 Diagnosis of abnormal human fertilization status based on pronuclear origin and/or centrosome number. J. Assist. Reprod. Genet. **32**, 1589-1595. (10.1007/s10815-015-0568-1) 26395191 PMC4651942

[102] Sathananthan AH, Kola I, Osborne J, Trounson A, Ng SC, Bongso A, Ratnam SS. 1991 Centrioles in the beginning of human development. Proc. Natl Acad. Sci. USA **88**, 4806-4810. (10.1073/pnas.88.11.4806) 2052559 PMC51755

[103] Uzbekov R, Singina GN, Shedova EN, Banliat C, Avidor-Reiss T, Uzbekova S. 2023 Centrosome formation in the bovine early embryo. Cells **12**, 1335. (10.3390/cells12091335) 37174735 PMC10177215

[104] Balestra FR et al. 2021 TRIM37 prevents formation of centriolar protein assemblies by regulating Centrobin. ELife **10**, e62640. (10.7554/eLife.62640) 33491649 PMC7870141

[105] Li J, Kim S, Kobayashi T, Liang F, Korzeniewski N, Duensing S, Dynlacht BD. 2012 Neurl4, a novel daughter centriole protein, prevents formation of ectopic microtubule organizing centres. EMBO Rep. **13**, 547-553. (10.1038/embor.2012.40) 22441691 PMC3367236

[106] Li S, Fernandez J-J, Marshall WF, Agard DA. 2012 Three-dimensional structure of basal body triplet revealed by electron cryo-tomography. EMBO J. **31**, 552-562. (10.1038/emboj.2011.460) 22157822 PMC3273388

[107] Meitinger F, Kong D, Ohta M, Desai A, Oegema K, Loncarek J. 2021 TRIM37 prevents formation of condensate-organized ectopic spindle poles to ensure mitotic fidelity. J. Cell Biol. **220**, 7. (10.1083/jcb.202010180) PMC8127006 33983387

[108] Shiratsuchi G, Takaoka K, Ashikawa T, Hamada H, Kitagawa D. 2015 RBM 14 prevents assembly of centriolar protein complexes and maintains mitotic spindle integrity. EMBO J. **34**, 97-114. (10.15252/embj.201488979) 25385835 PMC4291483

[109] Chang TB, Hsu JC-C, Yang TT. 2023 Single-molecule localization microscopy reveals the ultrastructural constitution of distal appendages in expanded mammalian centrioles. Nat. Commun. **14**, 1688. (10.1038/s41467-023-37342-x) 36973278 PMC10043031

[110] Woglar A, Pierron M, Schneider FZ, Jha K, Busso C, Gönczy P. 2022 Molecular architecture of the C. elegans centriole. PLoS Biol. **20**, e3001784. (10.1371/journal.pbio.3001784) 36107993 PMC9531800

[111] Dingle AD, Fulton C. 1966 Development of the flagellar apparatur of Naegleria. J. Cell Biol. **31**, 43-54. (10.1083/jcb.31.1.43) 5971974 PMC2107041

[112] Fulton C, Dingle AD. 1971 Basal bodies, but not centrioles, in Naegleria. J. Cell Biol. **51**, 826-836. (10.1083/jcb.51.3.826) 4942778 PMC2108039

[113] Mahowald AP, Strassheim JM. 1970 Intercellular migration of centrioles in the germarium of Drosophila melanogaster. J. Cell Biol. **45**, 306-320. (10.1083/jcb.45.2.306) 4327572 PMC2107913

[114] Mahowald AP, Caulton JH, Edwards MK, Floyd AD. 1979 Loss of centrioles and polyploidization in follicle cells of Drosophila melanogaster. Exp. Cell Res. **118**, 404-410. (10.1016/0014-4827(79)90167-8) 104872

[115] Riparbelli MG, Persico V, Gottardo M, Callaini G. 2018 The developing *Drosophila* eye: an oncoming model to study centriole reduction. J. Cell Sci. **131**, jcs211441. (10.1242/jcs.211441) 29361550

[116] Schoenfelder KP, Montague RA, Paramore SV, Lennox AL, Mahowald AP, Fox DT. 2014 Indispensable pre-mitotic endocycles promote aneuploidy in the Drosophila rectum. Development **141**, 3551-3560. (10.1242/dev.109850) 25142462 PMC6517832

[117] Zheng Y, Buchwalter RA, Zheng C, Wight EM, Chen JV, Megraw TL. 2020 A perinuclear microtubule-organizing centre controls nuclear positioning and basement membrane secretion. Nat. Cell Biol. **22**, 297-309. (10.1038/s41556-020-0470-7) 32066907 PMC7161059

[118] Mogensen MM, Tucker JB. 1987 Evidence for microtubule nucleation at plasma membrane-associated sites in *Drosophila*. J. Cell Sci. **88**, 95-107. (10.1242/jcs.88.1.95) 3127404

[119] Tassin AM, Maro B, Bornens M. 1985 Fate of microtubule organizing centers during in vitro myogenesis. J. Cell Biol. **100**, 35-46. (10.1083/jcb.100.1.35) 3880758 PMC2113478

[120] Bugnard E, Zaal KJM, Ralston E. 2005 Reorganization of microtubule nucleation during muscle differentiation. Cell Motility Cytoskelet. **60**, 1-13. (10.1002/cm.20042) 15532031

[121] Przybylski RJ. 1971 Occurrence of centrioles during skeletal and cardiac myogenesis. J. Cell Biol. **49**, 214-221. (10.1083/jcb.49.1.214) 5555575 PMC2108205

[122] Connolly JA, Kiosses BW, Kalnins VI. 1986 Centrioles are lost as embryonic myoblasts fuse into myotubes in vitro. Eur. J. Cell Biol. **39**, 341-345. 3514220

[123] Serwas D, Su TY, Roessler M, Wang S, Dammermann A. 2017 Centrioles initiate cilia assembly but are dispensable for maturation and maintenance in *C. elegans*. J. Cell Biol. **216**, 1659-1671. (10.1083/jcb.201610070) 28411189 PMC5461022

[124] Lu Y, Roy R. 2014 Centrosome/cell cycle uncoupling and elimination in the endoreduplicating intestinal cells of C. elegans. PLoS ONE **9**, e110958. (10.1371/journal.pone.0110958) 25360893 PMC4215990

[125] Kalbfuss N, Berger A, Gönczy P. 2023 Mapping of centriolar proteins onto the post-embryonic lineage of *C. elegans*. *BioRxiv*. (10.1101/2023.05.25.542230)

[126] Kalbfuss N, Gönczy P. 2023 Extensive programmed centriole elimination unveiled in C. elegans embryos. Sci. Adv. **9**, eadg8682. (10.1126/sciadv.adg8682) 37256957 PMC10413642

[127] Komarova IA, Vorob'ev IA. 1994 The ultrastructure of the cell center in the enterocytes of mouse embryos and newborn mice. Ontogenez **25**, 76-88. 8190453

[128] Woolley DM, Fawcett DW. 1973 The degeneration and disappearance of the centrioles during the development of the rat spermatozoon. Anat. Rec. **177**, 289-301. (10.1002/ar.1091770209) 4356969

[129] Phillips DM. 1970 Insect sperm: their structure and morphogenesis. J. Cell Biol. **44**, 243-277. (10.1083/jcb.44.2.243) 4903810 PMC2107952

[130] Garbrecht J, Laos T, Holzer E, Dillinger M, Dammermann A. 2021 An acentriolar centrosome at the C. elegans ciliary base. Curr. Biol. **31**, 2418-2428.e8. (10.1016/j.cub.2021.03.023) 33798427

[131] Magescas J, Eskinazi S, Tran MV, Feldman JL. 2021 Centriole-less pericentriolar material serves as a microtubule organizing center at the base of C. elegans sensory cilia. Curr. Biol. **31**, 2410-2417.e6. (10.1016/j.cub.2021.03.022) 33798428 PMC8277230

[132] Bezler A, Gönczy P. 2010 Mutual antagonism between the anaphase promoting complex and the spindle assembly checkpoint contributes to mitotic timing in Caenorhabditis elegans. Genetics **186**, 1271-1283. (10.1534/genetics.110.123133) 20944014 PMC2998310

[133] Hamill DR, Severson AF, Carter JC, Bowerman B. 2002 Centrosome maturation and mitotic spindle assembly in *C. elegans* require SPD-5, a protein with multiple coiled-coil domains. Dev. Cell **3**, 673-684. (10.1016/S1534-5807(02)00327-1) 12431374

[134] Buss G, Stratton MB, Milenkovic L, Stearns T. 2022 Postmitotic centriole disengagement and maturation leads to centrosome amplification in polyploid trophoblast giant cells. Mol. Biol. Cell **33**, ar118. (10.1091/mbc.E22-05-0182) 36001376 PMC9634975

[135] Delattre M, Leidel S, Wani K, Baumer K, Bamat J, Schnabel H, Feichtinger R, Schnabel R, Gönczy P. 2004 Centriolar SAS-5 is required for centrosome duplication in C. elegans. Nat. Cell Biol. **6**, 656-664. (10.1038/ncb1146) 15232593

[136] Shirato Y, Tamura M, Yoneda M, Nemoto S. 2006 Centrosome destined to decay in starfish oocytes. Development **133**, 343-350. (10.1242/dev.02193) 16368931

[137] Tamura M, Nemoto S. 2001 Reproductive Maternal Centrosomes Are Cast off into Polar Bodies during Maturation Division in Starfish Oocytes. Exp. Cell Res. **269**, 130-139. (10.1006/excr.2001.5305) 11525646

[138] Uetake Y, Kato KH, Washitani-Nemoto S, Nemoto S. 2002 Nonequivalence of maternal centrosomes/centrioles in starfish oocytes: selective casting-off of reproductive centrioles into polar bodies. Dev. Biol. **247**, 149-164. (10.1006/dbio.2002.0682) 12074559

[139] Zhang QY, Tamura M, Uetake Y, Washitani-Nemoto S, Nemoto S. 2004 Regulation of the paternal inheritance of centrosomes in starfish zygotes. Dev. Biol. **266**, 190-200. (10.1016/j.ydbio.2003.10.027) 14729488

[140] Boveri T. 1914 Zur frage der entstehung maligner tumoren. Jena, Germany: Gustav Fischer Verlag.

[141] Snook RR, Hosken DJ, Karr TL. 2011 The biology and evolution of polyspermy: insights from cellular and functional studies of sperm and centrosomal behavior in the fertilized egg. Reproduction **142**, 779-792. (10.1530/REP-11-0255) 21964827

[142] Maller J, Poccia D, Nishioka D, Kidd P, Gerhart J, Hartman H. 1976 Spindle formation and cleavage in Xenopus eggs injected with centriole-containing fractions from sperm. Exp. Cell Res. **99**, 285-294. (10.1016/0014-4827(76)90585-1) 944633

[143] Tournier F, Karsenti E, Bornens M. 1989 Parthenogenesis in Xenopus eggs injected with centrosomes from synchronized human lymphoid cells. Dev. Biol. **136**, 321-329. (10.1016/0012-1606(89)90259-5) 2583369

[144] Tournier F, Komesli S, Paintrand M, Job D, Bornens M. 1991 The intercentriolar linkage is critical for the ability of heterologous centrosomes to induce parthenogenesis in Xenopus. J. Cell Biol. **113**, 1361-1369. (10.1083/jcb.113.6.1361) 2045416 PMC2289023

[145] Sanchez AD, Feldman JL. 2017 Microtubule-organizing centers: from the centrosome to non-centrosomal sites. Curr. Opin. Cell Biol. **44**, 93-101. (10.1016/j.ceb.2016.09.003) 27666167 PMC5362366

[146] Mikule K, Delaval B, Kaldis P, Jurcyzk A, Hergert P, Doxsey S. 2007 Loss of centrosome integrity induces p38–p53–p21-dependent G1–S arrest. Nat. Cell Biol. **9**, 160-170. (10.1038/ncb1529) 17330329

[147] Kochanski RS, Borisy GG. 1990 Mode of centriole duplication and distribution. J. Cell Biol. **110**, 1599-1605. (10.1083/jcb.110.5.1599) 2335566 PMC2200183

[148] Conduit PT, Raff JW. 2010 Cnn dynamics drive centrosome size asymmetry to ensure daughter centriole retention in Drosophila neuroblasts. Curr. Biol. **20**, 2187-2192. (10.1016/j.cub.2010.11.055) 21145745

[149] Januschke J, Llamazares S, Reina J, Gonzalez C. 2011 Drosophila neuroblasts retain the daughter centrosome. Nat. Commun. **2**, 243. (10.1038/ncomms1245) 21407209 PMC3072095

[150] Salzmann V, Chen C, Chiang C-YA, Tiyaboonchai A, Mayer M, Yamashita YM. 2014 Centrosome-dependent asymmetric inheritance of the midbody ring in Drosophila germline stem cell division. Mol. Biol. Cell **25**, 267-275. (10.1091/mbc.e13-09-0541) 24227883 PMC3890347

[151] Yamashita YM, Mahowald AP, Perlin JR, Fuller MT. 2007 Asymmetric inheritance of mother versus daughter centrosome in stem cell division. Science **315**, 518-521. (10.1126/science.1134910) 17255513 PMC2563045

[152] Gilbert SF. 2000 Developmental biology: early Drosophila development, 6th edn. Sunderland, MA: Sinauer Associates.

[153] Palazzo RE, Vaisberg E, Cole RW, Rieder CL. 1992 Centriole duplication in lysates of *Spisula solidissima* oocytes. Science **256**, 219-221. (10.1126/science.1566068) 1566068

[154] Pelletier L, O'Toole E, Schwager A, Hyman AA, Müller-Reichert T. 2006 Centriole assembly in Caenorhabditis elegans. Nature **444**, 619-623. (10.1038/nature05318) 17136092

[155] Kong D, Sahabandu N, Sullenberger C, Vásquez-Limeta A, Luvsanjav D, Lukasik K, Loncarek J. 2020 Prolonged mitosis results in structurally aberrant and over-elongated centrioles. J. Cell Biol. **219**, e201910019. 10.1083/jcb.201910019 32271878 PMC7265320

[156] Kuriyama R, Borisy GG. 1981 Centriole cycle in Chinese hamster ovary cells as determined by whole-mount electron microscopy. J. Cell Biol. **91**, 814-821. (10.1083/jcb.91.3.814) 7328123 PMC2112828

[157] He L, Wang X, Montell DJ. 2011 Shining light on Drosophila oogenesis: live imaging of egg development. Curr. Opin. Genet. Dev. **21**, 612-619. (10.1016/j.gde.2011.08.011) 21930372 PMC6824908

[158] Li W, Yi P, Zhu Z, Zhang X, Li W, Ou G. 2017 Centriole translocation and degeneration during ciliogenesis in *Caenorhabditis elegans* neurons. EMBO J. **36**, 2553-2566. (10.15252/embj.201796883) 28743734 PMC5579362

[159] Mikeladze-Dvali T, von Tobel L, Strnad P, Knott G, Leonhardt H, Schermelleh L, Gonczy P. 2012 Analysis of centriole elimination during C. elegans oogenesis. Development **139**, 1670-1679. (10.1242/dev.075440) 22492357 PMC4074223

[160] Iwao Y, Kimoto C, Fujimoto A, Suda A, Hara Y. 2020 Physiological polyspermy: selection of a sperm nucleus for the development of diploid genomes in amphibians. Mol. Reprod. Dev. **87**, 358-369. (10.1002/mrd.23235) 31310413

[161] Carré D, Sardet C. 1984 Fertilization and early development in Beroe ovata. Dev. Biol. **105**, 188-195. (10.1016/0012-1606(84)90274-4) 6147287

[162] Iwao Y, Murakawa T, Yamaguchi J, Yamashita M. 2002 Localization of gamma-tubulin and cyclin B during early cleavage in physiologically polyspermic newt eggs. Dev. Growth Diff. **44**, 489-499. (10.1046/j.1440-169X.2002.00661.x) 12492507

[163] Rouvière C, Houliston E, Carré D, Chang P, Sardet C. 1994 Characteristics of pronuclear migration in *Beroe ovata*. Cell Motility Cytoskeleton **29**, 301-311. (10.1002/cm.970290403) 7859293

[164] Iwao Y, Elinson RP. 1990 Control of sperm nuclear behavior in physiologically polyspermic newt eggs: possible involvement of MPF. Dev. Biol. **142**, 301-312. (10.1016/0012-1606(90)90351-I) 2257969

[165] Nandi D, Tahiliani P, Kumar A, Chandu D. 2006 The ubiquitin-proteasome system. J. Biosci. **31**, 137-155. (10.1007/BF02705243) 16595883

[166] Inoué S, Sato H. 1967 Cell motility by labile association of molecules. The nature of mitotic spindle fibers and their role in chromosome movement. J. Gen. Physiol. **50**, 259-292. PMC2225745 6058222

[167] Kasahara K, Inagaki M. 2021 Primary ciliary signaling: links with the cell cycle. Trends Cell Biol. **31**, 954-964. (10.1016/j.tcb.2021.07.009) 34420822

[168] Fechter J, Schöneberg A, Schatten G. 1996 Excision and disassembly of sperm tail microtubules during sea urchin fertilization: requirements for microtubule dynamics. Cell Motility Cytoskeleton **35**, 281-288. (10.1002/(SICI)1097-0169(1996)35:4<281::AID-CM1>3.0.CO;2-A) 8956000

[169] Saxton WM, Stemple DL, Leslie RJ, Salmon ED, Zavortink M, McIntosh JR. 1984 Tubulin dynamics in cultured mammalian cells. J. Cell Biol. **99**, 2175-2186. (10.1083/jcb.99.6.2175) 6501419 PMC2113582

[170] Schulze E, Kirschner M. 1986 Microtubule dynamics in interphase cells. J. Cell Biol. **102**, 1020-1031. (10.1083/jcb.102.3.1020) 3512576 PMC2114101

[171] Wong YL et al. 2015 Reversible centriole depletion with an inhibitor of Polo-like kinase 4. Science **348**, 1155-1160. (10.1126/science.aaa5111) 25931445 PMC4764081

[172] Balestra FR, Tobel LV, Gönczy P. 2015 Paternally contributed centrioles exhibit exceptional persistence in *C. elegans* embryos. Cell Res. **25**, 642-644. (10.1038/cr.2015.49) 25906994 PMC4423087

[173] Guichard P et al. 2013 Native architecture of the centriole proximal region reveals features underlying its 9-fold radial symmetry. Curr. Biol. **23**, 1620-1628. (10.1016/j.cub.2013.06.061) 23932403

[174] Dutcher SK, Morrissette NS, Preble AM, Rackley C, Stanga J. 2002 ε-Tubulin is an essential component of the centriole. Mol. Biol. Cell **13**, 3859-3869. (10.1091/mbc.e02-04-0205) 12429830 PMC133598

[175] Dutcher SK, Trabuco EC. 1998 The *UNI3* gene is required for assembly of basal bodies of *Chlamydomonas* and encodes δ-tubulin, a new member of the tubulin superfamily. Mol. Biol. Cell **9**, 1293-1308. (10.1091/mbc.9.6.1293) 9614175 PMC25351

[176] Breslow DK et al. 2018 A CRISPR-based screen for Hedgehog signaling provides insights into ciliary function and ciliopathies. Nat. Genet. **50**, 460-471. (10.1038/s41588-018-0054-7) 29459677 PMC5862771

[177] Le Clech, M. 2008 Role of CAP350 in centriolar tubule stability and centriole assembly. PLoS ONE **3**, e3855. (10.1371/journal.pone.0003855) 19052644 PMC2586089

[178] Kilburn CL, Pearson CG, Romijn EP, Meehl JB, Giddings TH, Culver BP, Yates JR, Winey M. 2007 New Tetrahymena basal body protein components identify basal body domain structure. J. Cell Biol. **178**, 905-912. (10.1083/jcb.200703109) 17785518 PMC2064616

[179] Pearson CG, Osborn DPS, Giddings TH, Beales PL, Winey M. 2009 Basal body stability and ciliogenesis requires the conserved component Poc1. J. Cell Biol. **187**, 905-920. (10.1083/jcb.200908019) 20008567 PMC2806327

[180] Venoux M, Tait X, Hames RS, Straatman KR, Woodland HR, Fry AM. 2013 Poc1A and Poc1B act together in human cells to ensure centriole integrity. J. Cell Sci. **126**, 163-175. (10.1242/jcs.111203) 23015594 PMC3603514

[181] Gudi R, Zou C, Li J, Gao Q. 2011 Centrobin-tubulin interaction is required for centriole elongation and stability. J. Cell Biol. **193**, 711-725. (10.1083/jcb.201006135) 21576394 PMC3166857

[182] Zou C, Li J, Bai Y, Gunning WT, Wazer DE, Band V, Gao Q. 2005 Centrobin: a novel daughter centriole-associated protein that is required for centriole duplication. J. Cell Biol. **171**, 437-445. (10.1083/jcb.200506185) 16275750 PMC2171251

[183] Gudi R, Haycraft CJ, Bell PD, Li Z, Vasu C. 2015 Centrobin-mediated regulation of the centrosomal protein 4.1-associated protein (CPAP) level limits centriole length during elongation stage. J. Biol. Chem. **290**, 6890-6902. (10.1074/jbc.M114.603423) 25616662 PMC4358114

[184] Matsuura K, Lefebvre PA, Kamiya R, Hirono M. 2004 Bld10p, a novel protein essential for basal body assembly in Chlamydomonas. J. Cell Biol. **165**, 663-671. (10.1083/jcb.200402022) 15173189 PMC2172387

[185] Bayless BA, Giddings TH, Winey M, Pearson CG. 2012 Bld10/Cep135 stabilizes basal bodies to resist cilia-generated forces. Mol. Biol. Cell **23**, 4820-4832. (10.1091/mbc.e12-08-0577) 23115304 PMC3521689

[186] Jakobsen L et al. 2011 Novel asymmetrically localizing components of human centrosomes identified by complementary proteomics methods. EMBO J. **30**, 1520-1535. (10.1038/emboj.2011.63) 21399614 PMC3102290

[187] Gönczy P, Schnabel H, Kaletta T, Amores AD, Hyman T, Schnabel R. 1999 Dissection of cell division processes in the one cell stage *Caenorhabditis elegans* embryo by mutational analysis. J. Cell Biol. **144**, 927-946. (10.1083/jcb.144.5.927) 10085292 PMC2148205

[188] von Tobel L, Mikeladze-Dvali T, Delattre M, Balestra FR, Blanchoud S, Finger S, Knott G, Müller-Reichert T, Gönczy P. 2014 SAS-1 is a C2 domain protein critical for centriole integrity in *C. elegans*. PLoS Genetics **10**, e1004777. (10.1371/journal.pgen.1004777) 25412110 PMC4238951

[189] Balestra FR, Strnad P, Flückiger I, Gönczy P. 2013 Discovering regulators of centriole biogenesis through siRNA-based functional genomics in human cells. Dev. Cell **25**, 555-571. (10.1016/j.devcel.2013.05.016) 23769972

[190] Thauvin-Robinet C et al. 2014 The oral-facial-digital syndrome gene *C2CD3* encodes a positive regulator of centriole elongation. Nat. Genet. **46**, 905-911. (10.1038/ng.3031) 24997988 PMC4120243

[191] Hoover AN, Wynkoop A, Zeng H, Jia J, Niswander LA, Liu A. 2008 C2cd3 is required for cilia formation and Hedgehog signaling in mouse. Development **135**, 4049-4058. (10.1242/dev.029835) 19004860 PMC3120044

[192] Gaudin N et al. 2022 Evolutionary conservation of centriole rotational asymmetry in the human centrosome. ELife **11**, e72382. (10.7554/eLife.72382) 35319462 PMC8983040

[193] Janke C, Magiera MM. 2020 The tubulin code and its role in controlling microtubule properties and functions. Nat. Rev. Mol. Cell Biol. **21**, 307-326. (10.1038/s41580-020-0214-3) 32107477

[194] Bobinnec Y, Khodjakov A, Mir LM, Rieder CL, Eddé B, Bornens M. 1998 Centriole disassembly in vivo and its effect on centrosome structure and function in vertebrate cells. J. Cell Biol. **143**, 1575-1589. (10.1083/jcb.143.6.1575) 9852152 PMC2132987

[195] Clift D, McEwan WA, Labzin LI, Konieczny V, Mogessie B, James LC, Schuh M. 2017 A method for the acute and rapid degradation of endogenous proteins. Cell **171**, 1692-1706.e18. (10.1016/j.cell.2017.10.033) 29153837 PMC5733393

[196] Goshima G, Wollman R, Goodwin SS, Zhang N, Scholey JM, Vale RD, Stuurman N. 2007 Genes required for mitotic spindle assembly in *Drosophila* S2 cells. Science **316**, 417-421. (10.1126/science.1141314) 17412918 PMC2837481

[197] Neumann B et al. 2010 Phenotypic profiling of the human genome by time-lapse microscopy reveals cell division genes. Nature **464**, 721-727. (10.1038/nature08869) 20360735 PMC3108885

[198] Gönczy P et al. 2000 Functional genomic analysis of cell division in C. elegans using RNAi of genes on chromosome III. Nature **408**, 331-336. (10.1038/35042526) 11099034

[199] Kamath RS, Martinez-Campos M, Zipperlen P, Fraser AG, Ahringer J. 2001 Effectiveness of specific RNA-mediated interference through ingested double-stranded RNA in Caenorhabditis elegans. Genome Biol. **2**, RESEARCH0002. (10.1186/gb-2000-2-1-research0002) 11178279 PMC17598

[200] Kamath RS, Ahringer J. 2003 Genome-wide RNAi screening in Caenorhabditis elegans. Methods **30**, 313-321. (10.1016/S1046-2023(03)00050-1) 12828945

[201] O'Connell KF, Leys CM, White JG. 1998 A genetic screen for temperature-sensitive cell-division mutants of *Caenorhabditis elegans*. Genetics **149**, 1303-1321. (10.1093/genetics/149.3.1303) 9649522 PMC1460235

[202] O'Connell KF, Caron C, Kopish KR, Hurd DD, Kemphues KJ, Li Y, White JG. 2001 The C. elegans zyg-1 gene encodes a regulator of centrosome duplication with distinct maternal and paternal roles in the embryo. Cell **105**, 547-558. (10.1016/S0092-8674(01)00338-5) 11371350

[203] Sönnichsen B et al. 2005 Full-genome RNAi profiling of early embryogenesis in Caenorhabditis elegans. Nature **434**, 462-469. (10.1038/nature03353) 15791247

[204] Matsuura R, Ashikawa T, Nozaki Y, Kitagawa D. 2016 LIN-41 inactivation leads to delayed centrosome elimination and abnormal chromosome behavior during female meiosis in Caenorhabditis elegans. Mol. Biol. Cell **27**, 799-811. (10.1091/mbc.E15-10-0713) 26764090 PMC4803306

[205] Boag PR, Atalay A, Robida S, Reinke V, Blackwell TK. 2008 Protection of specific maternal messenger RNAs by the P body protein CGH-1 (Dhh1/RCK) during Caenorhabditis elegans oogenesis. J. Cell Biol. **182**, 543-557. (10.1083/jcb.200801183) 18695045 PMC2500139

[206] Shang Y, Li B, Gorovsky MA. 2002 Tetrahymena thermophila contains a conventional γ-tubulin that is differentially required for the maintenance of different microtubule-organizing centers. J. Cell Biol. **158**, 1195-1206. (10.1083/jcb.200205101) 12356864 PMC2173235

[207] Schweizer N, Haren L, Dutto I, Viais R, Lacasa C, Merdes A, Lüders J. 2021 Sub-centrosomal mapping identifies augmin-γTuRC as part of a centriole-stabilizing scaffold. Nat. Commun. **12**, 6042. (10.1038/s41467-021-26252-5) 34654813 PMC8519919

[208] Harper NC, Rillo R, Jover-Gil S, Assaf ZJ, Bhalla N, Dernburg AF. 2011 Pairing centers recruit a Polo-like kinase to orchestrate meiotic chromosome dynamics in *C. elegans*. Dev. Cell **21**, 934-947. (10.1016/j.devcel.2011.09.001) 22018922 PMC4343031

[209] Pimenta-Marques A, Perestrelo T, Rodrigues P, Duarte P, Lince-Faria M, Bettencourt-Dias M. 2022 Ana1/CEP295 is an essential player in the centrosome maintenance program regulated by Polo kinase. *BioRxiv*. (10.1101/2022.04.06.487296) PMC10897187 38200359

[210] Fritz-Laylin LK, Fulton C. 2016 Naegleria: a classic model for de novo basal body assembly. Cilia **5**, 10. (10.1186/s13630-016-0032-6) 27047659 PMC4819266

[211] Azimzadeh J. 2014 Exploring the evolutionary history of centrosomes. Phil. Trans. R. Soc. B **369**, 20130453. (10.1098/rstb.2013.0453) 25047607 PMC4113097

[212] Carvalho-Santos Z, Azimzadeh J, Pereira-Leal JB, Bettencourt-Dias M. 2011 Tracing the origins of centrioles, cilia, and flagella. J. Cell Biol. **194**, 165-175. (10.1083/jcb.201011152) 21788366 PMC3144413

[213] Satir P, Guerra C, Bell AJ. 2007 Evolution and persistence of the cilium. Cell Motility Cytoskeleton **64**, 906-913. (10.1002/cm.20238) 17896340

[214] Yamauchi Y, Greber UF. 2016 Principles of virus uncoating: cues and the snooker ball. Traffic **17**, 569-592. (10.1111/tra.12387) 26875443 PMC7169695

[215] Strunze S et al. 2011 Kinesin-1-mediated capsid disassembly and disruption of the nuclear pore complex promote virus infection. Cell Host Microbe **10**, 210-223. (10.1016/j.chom.2011.08.010) 21925109

[216] Kulanayake S, Tikoo S. 2021 Adenovirus core proteins: structure and function. Viruses **13**, 388. (10.3390/v13030388) 33671079 PMC7998265

[217] Bauer M, Flatt JW, Seiler D, Cardel B, Emmenlauer M, Boucke K, Suomalainen M, Hemmi S, Greber UF. 2019 The E3 ubiquitin ligase mind bomb 1 controls adenovirus genome release at the nuclear pore complex. Cell Rep. **29**, 3785-3795.e8. (10.1016/j.celrep.2019.11.064) 31851912

[218] Ploubidou A, Moreau V, Ashman K, Reckmann I, González C, Way M. 2000 Vaccinia virus infection disrupts microtubule organization and centrosome function. EMBO J. **19**, 3932-3944. (10.1093/emboj/19.15.3932) 10921875 PMC306617

[219] Bedoui S, Herold MJ, Strasser A. 2020 Emerging connectivity of programmed cell death pathways and its physiological implications. Nat. Rev. Mol. Cell Biol. **21**, 678-695. (10.1038/s41580-020-0270-8) 32873928

[220] Nössing C, Ryan KM. 2023 50 years on and still very much alive: ‘Apoptosis: a basic biological phenomenon with wide-ranging implications in tissue kinetics'. Br. J. Cancer **128**, 426-431. (10.1038/s41416-022-02020-0) 36369364 PMC9938139

[221] Levine MS et al. 2017 Centrosome amplification is sufficient to promote spontaneous tumorigenesis in mammals. Dev. Cell **40**, 313-322.e5. (10.1016/j.devcel.2016.12.022) 28132847 PMC5296221

[222] Marteil G et al. 2018 Over-elongation of centrioles in cancer promotes centriole amplification and chromosome missegregation. Nat. Commun. **9**, 1258. (10.1038/s41467-018-03641-x) 29593297 PMC5871873

[223] Serçin Ö et al. 2016 Transient PLK4 overexpression accelerates tumorigenesis in p53-deficient epidermis. Nat. Cell Biol. **18**, 100-110. (10.1038/ncb3270) 26595384

[224] Khodjakov A, Rieder CL. 2001 Centrosomes enhance the fidelity of cytokinesis in vertebrates and are required for cell cycle progression. J. Cell Biol. **153**, 237-242. (10.1083/jcb.153.1.237) 11285289 PMC2185537

[225] Lambrus BG, Uetake Y, Clutario KM, Daggubati V, Snyder M, Sluder G, Holland AJ. 2015 p53 protects against genome instability following centriole duplication failure. J. Cell Biol. **210**, 63-77. (10.1083/jcb.201502089) 26150389 PMC4494000

[226] Sir J-H, Pütz M, Daly O, Morrison CG, Dunning M, Kilmartin JV, Gergely F. 2013 Loss of centrioles causes chromosomal instability in vertebrate somatic cells. J. Cell Biol. **203**, 747-756. (10.1083/jcb.201309038) 24297747 PMC3857480

[227] Wang M, Nagle RB, Knudsen BS, Cress AE, Rogers GC. 2020 Centrosome loss results in an unstable genome and malignant prostate tumors. Oncogene **39**, 399-413. (10.1038/s41388-019-0995-z) 31477840

